# Wrapping anisotropic microgel particles in lipid membranes: Effects of particle shape and membrane rigidity

**DOI:** 10.1073/pnas.2217534120

**Published:** 2023-07-17

**Authors:** Xiaoyan Liu, Thorsten Auth, Nabanita Hazra, Morten Frendø Ebbesen, Jonathan Brewer, Gerhard Gompper, Jérôme J. Crassous, Emma Sparr

**Affiliations:** ^a^School of Chemistry and Chemical Engineering, Shaanxi Normal University, Xi’an 710062, China; ^b^Division of Physical Chemistry, Department of Chemistry, Lund University, Lund 22100, Sweden; ^c^Theoretical Physics of Living Matter, Institute of Biological Information Processing and Institute for Advanced Simulation, Forschungszentrum Jülich, Jülich 52428 Germany; ^d^Institute of Physical Chemistry, Rheinisch-Westfälische Technische Hochschule Aachen, Aachen 52074, Germany; ^e^Department of Biochemistry and Molecular Biology, University of Southern Denmark, Odense 5230, Denmark

**Keywords:** lipid membrane, giant unilamellar vesicles, anisotropic microgels, wrapping, membrane adhesion

## Abstract

Cellular engulfment and uptake of macromolecular assemblies or nanoparticles via endocytosis can be associated to both healthy and disease-related biological processes as well as delivery of drug nanoparticles and potential nanotoxicity of pollutants. Depending on the physical and chemical properties of the system, the adsorbed particles may remain at the membrane surface, become wrapped by the membrane, or translocate across the membrane through an endocytosis-like process. In this paper, we address the question of how the wrapping of colloidal particles by lipid membranes can be controlled by the shape of the particles, the particle–membrane adhesion energy, the membrane phase behavior, and the membrane-bending rigidity. We use a model system composed of soft core–shell microgel particles with spherical and ellipsoidal shapes, together with phospholipid membranes with varying composition. Confocal microscopy data clearly demonstrate how tuning of these basic properties of particles and membranes can be used to direct wrapping and membrane deformation and the organization of the particles at the membrane. The deep-wrapped states are more favorable for ellipsoidal than for spherical microgel particles of similar volume. Theoretical calculations for fixed adhesion strength predict the opposite behavior—wrapping becomes more difficult with increasing aspect ratio. The comparison with the experiments implies that the microgel adhesion strength must increase with increasing particle stretching. Considering the versatility offered by microgels systems to be synthesized with different shapes, functionalizations, and mechanical properties, the present findings further inspire future studies involving nanoparticle–membrane interactions relevant for the design of novel biomaterials and therapeutic applications.

## Significance

The cellular uptake of colloidal-sized particles of biological or synthetic origin has important implications for cellular function, and for the design of particles for diagnostic and therapeutic applications in nanomedicine. Here, we present experimental data combined with theoretical modeling showing how anisotropic microgels wrap at the lipid membrane depending on the physicochemical properties of the particles and the membrane. Important properties are the bending rigidity of the membrane, the particle shape, and the adhesion energy between the particles and the membrane. Accounting for the possibility offered by microgel systems to be custom-designed, it further opens up opportunities for future fundamental studies, therapeutic applications, and self-assembly strategies which involve nanoparticle–membrane interactions.

Cellular engulfment and uptake of macromolecular assemblies or nanoparticles via endocytosis can be associated to both healthy and disease-related biological processes (1, 2) as well as delivery of drug nanoparticles and potential nanotoxicity of pollutants (3–5). In nanotechnological, biotechnological, and pharmaceutical applications (6–11), relevant examples of nonspherical assemblies exist, for example, virus capsids (12), discoidal high-density lipoprotein coassemblies (13), and antigen particles of various shapes (14). In vivo, endocytosis includes several subprocesses, with an intricate interplay between different molecular mechanisms that are close to impossible to decouple in studies of molecularly complex biological systems. A deepened understanding of these generic physical-chemical mechanisms that control the wrapping of particles by a lipid membrane can be reached through combined experimental and theoretical studies of model systems, where the physical and chemical parameters are controlled and systematically varied. Versatile colloidal model particles open up the possibility for systematic studies of the impact of particle shape and deformability on their wrapping by membranes.

Over the past two decades, theory and computer simulations have been extensively applied to predict the interactions between biological membranes and particles with spherical (15–29) or nonspherical (23, 30–37) shape. However, experimental studies so far have mainly focused on spherical (rigid and deformable) particles (15, 38–46). From these studies, it has been concluded that the membrane–particle interactions can be tuned by changing particle size and surface chemistry, particle and membrane deformability, as well as lipid acyl-chain order and membrane tension (39, 45). Studies using hard nonspherical particles indicate that particle shape is another important factor controlling particle translocation across a membrane, although the actual effects of particle geometry seem to vary between different particle systems (6, 7, 47–54). The possible inconsistency between some of these previous studies may be related to the difficulty in designing a set of particles that differ in shape but not in any other respect, such as volume and surface chemistry.

Microgel systems are particularly appealing as model systems because they offer many possibilities for the synthesis of different sizes, shapes, softness, and functionalities (55, 56). Size and softness of spherical microgels have been found to significantly alter cellular uptake, with either an increase with the particle rigidity (57) or a decrease with increased microgel cross-linking (58–60). In particular, small and soft poly(N-isopropylacrylamide) (PNIPAM) and poly(N-isopropylmethacrylamide) (PNIPMAM) microgels were found to present a faster internalization than large and rigid ones that may involve different internalization mechanisms depending on their size and rigidity (58–60). PNIPAM microgels below their volume phase transition temperature were observed to be partially wrapped and densely assembled at giant unilamellar vesicles (GUVs) in the fluid lipid phase (45, 46) and to be sparsely adsorbed in the gel phase (45). Nonspherical colloidal particles have been synthesized and characterized in bulk, including, ellipsoids (6, 7, 61, 62), rods (47), planes (7), soft nonspherical elastic particles, such as ellipsoidal microgels (61, 62), elliptical clusters (63), and anisotropic polymeric nanoparticles (64). However, experimental studies focusing on the conditions of the wrapping of anisotropic soft particles at lipid membranes are still lacking.

In this work, we address the question of how the wrapping of core–shell microgels by lipid membranes is affected by their shape, their membrane adhesion, and the membrane-bending rigidity. We do so by designing soft core–shell microgel particles with spherical and ellipsoidal shapes, together with a systematic variation of their membrane adhesion and the membrane-bending rigidity. The ellipsoidal microgel particles, which consist of a polystyrene core surrounded by a cross-linked PNIPMAM shell, were prepared from the same spherical “mother particles” by uniaxial stretching, thus resulting in soft particles with different shapes but almost identical volumes (62, 65). As a model membrane system, we use GUVs (66) composed of different zwitterionic phospholipids, where we vary the acyl chain composition, while keeping the lipid headgroup chemistry the same. We investigate particle adsorption and structural arrangement at the membrane in situ by confocal fluorescence microscopy. In particular, we study how the particles adsorb and organize at the vesicle surface and whether they get wrapped by the lipid membrane depending on their shape, their adhesion to the membrane, and the membrane-bending rigidity. We further analyze the energetics of wrapping for the same model systems in numerical calculations for ellipsoidal particles by variation of membrane-bending energy, membrane tension, and particle–membrane adhesion energy. Taken together, the combined experimental and theoretical analysis adds to the understanding of how particle and membrane physicochemical properties can influence engulfment and endocytosis processes in living cells. It further opens up the possibility for future studies addressing the influence of colloid softness, size, permeability, and functionality using custom-designed microgel systems.

## Results

### Particle Surface Adsorption and Wrapping in Lipid Membranes.

**Fig. 1.**
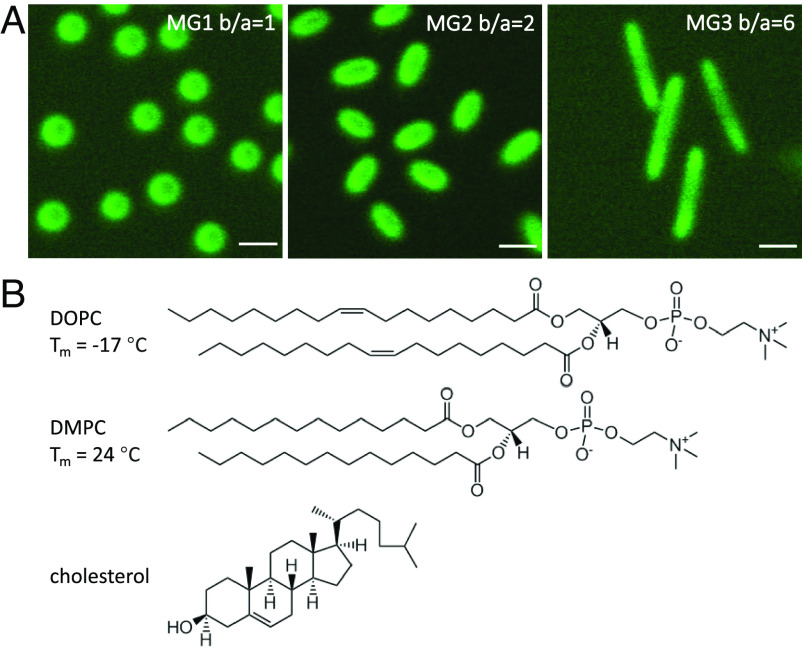
(*A*) 2D confocal laser scanning microscopy (CLSM) images of microgels, MG1 (spherical, aspect ratio b/a = 1), MG2 (ellipsoidal, b/a = 2), and MG3 (ellipsoidal, b/a = 6), at the glass coverslip. Temperature: 28 ^°^C. The scale bars represent 1 μm. (*B*) Molecular structures and melting temperatures (*T*_*m*_) of DOPC and DMPC lipids and molecular structure of cholesterol.

We study the adsorption and wrapping of microgel particles with spherical and ellipsoidal shapes on model membranes with different lipid compositions. The confocal images of the different microgel particles postlabeled with the green fluorescent probe Alexa488 are shown in Fig. 1 and *SI Appendix*, Fig. S3. The ellipsoidal microgel particles were prepared from the same spherical core–shell “mother microgel particles” with a core radius of 215 ± 13 nm and a hydrodynamic radius of 462 nm at 20 ^°^C (*SI Appendix*, Figs. S1–S4 and Table S1). This implies that the microgels have roughly the same volume but different shapes and surface areas. The translational diffusion and electrophoretic mobility measurements (*SI Appendix*, Table S1) indicate that the spherical and ellipsoidal microgels are positively charged. The particles of different shapes exhibit small differences in surface properties as inferred from ANS fluorescence that is sensitive to surface hydrophobicity (*SI Appendix*, Fig. S5) and electrophoretic mobility (*SI Appendix*, Table S1).

The lipid model membranes were formed by zwitterionic phosphatidylcholine (PC): either 1,2-dioleoyl-sn-glycero-3-phosphocholine (DOPC), 1,2-ditetradecanoyl-sn-glycero-3-phosphocholine (DMPC), or mixtures of DMPC and cholesterol (Fig. 1*B*), which for the present temperature conditions form either a liquid-disordered bilayer phase (DOPC and DMPC) or a liquid-ordered bilayer phase (DMPC/chol). These model membranes differ both with respect to their acyl-chain order and bending rigidity, while the lipid headgroup chemistry is the same for all systems. The bending rigidity of the liquid-ordered DMPC/chol bilayer is more than threefold that of the liquid-disordered DOPC and DMPC bilayers, where the latter are similar to each other. Based on fluctuation analysis in GUVs, the membrane-bending rigidity has been measured to *κ*_(DMPC/chol)_ ≈ 97 k_*B*_T (67, 68), *κ*_(DMPC)_ = 29–31 k_*B*_T (67–69), and *κ*_(DOPC)_ = 19–26 k_*B*_T (70–72) (T = 25–30 ^°^C). We note that the reported values of *κ* vary between different studies based on different techniques and model systems. For example, the analysis based on X-ray scattering of multilamellar stacks gives lower values of *κ*_(DMPC)_ (73) compared to the values obtained from the analysis of GUVs (67–69). The present model membranes also differ with respect to the apparent interfacial hydrophobicity due to their differences in the acyl-chain packing. The effective headgroup area in the liquid-disordered bilayer is larger for DOPC with its unsaturated chains [ca 70 Å^2^, (74)] as compared to fully saturated DMPC [ca 60 Å^2^, (74)]. The addition of cholesterol to the DMPC bilayer has a condensing effect on the acyl-chains (75), leading to further reduction in the effective area per lipid (PC and cholesterol) in the bilayer plane, although the effective area per PC headgroup is still similar to that in liquid-disordered PC bilayer (76). As the packing of the lipid acyl-chains in the bilayer is coupled both to the effective lipid headgroup area and the membrane rigidity, these physical properties of the membrane have to be evaluated together in relation to the particle adhesion and wrapping.

**Fig. 2.**
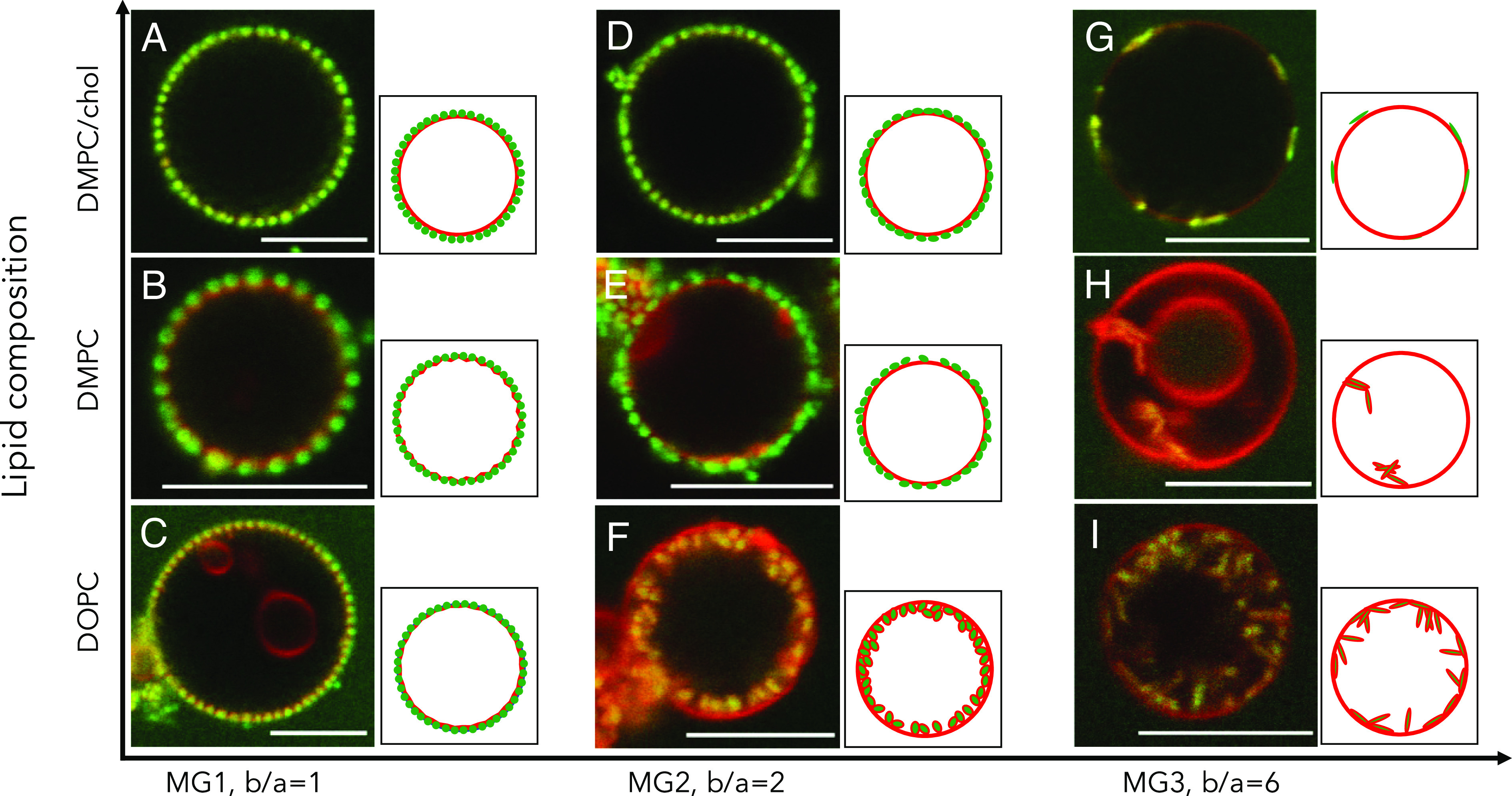
2D CLSM images of adsorption and wrapping of microgel particles (green) on lipid membrane (red) in GUVs. The microgel particles were either spherical MG1 (*A*–*C*) or ellipsoidal with different aspect ratios (b/a), MG2 (*D*–*F*) and MG3 (*G*–*I*), and the lipid composition was DMPC/chol (*A*, *D*, and *G*), DMPC (*B*, *E*, and *H*), and DOPC (*C*, *F*, and *I*). The lipid membrane differs both with respect to the membrane-bending rigidity, with the highest rigidity for DMPC/chol, and with respect to the effective area per headgroup, with the largest area for DOPC (67–72, 74, 76).

**Fig. 3.**
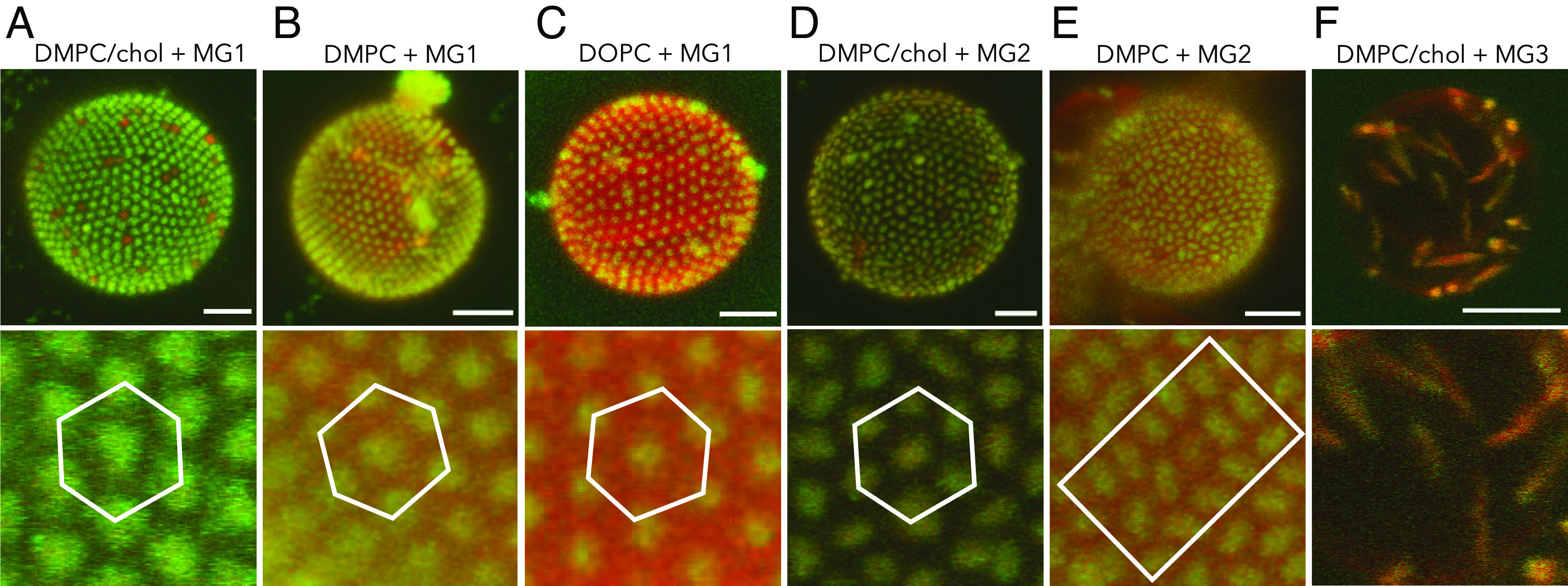
*Upper*: 3D CLSM images of the adsorbed spherical MG1 microgels (*A*–*C*), ellipsoidal MG2 (*D* and *E*), and MG3 (*F*) microgels on the GUVs composed of DMPC/chol (*A*, *D*, and *F*), DMPC (*B* and *E*), and DOPC (*C*). The 3D images were reconstructed from confocal *z*-stack images from the merged channels from microgels (green) and membranes labeled with Liss Rhod PE (red). *Lower*: The corresponding zoom-in images show the assembling structures of the microgels on the lipid membranes. Temperature: 28 ^°^C. (Scale bars, 5 μm.)

**Fig. 4.**
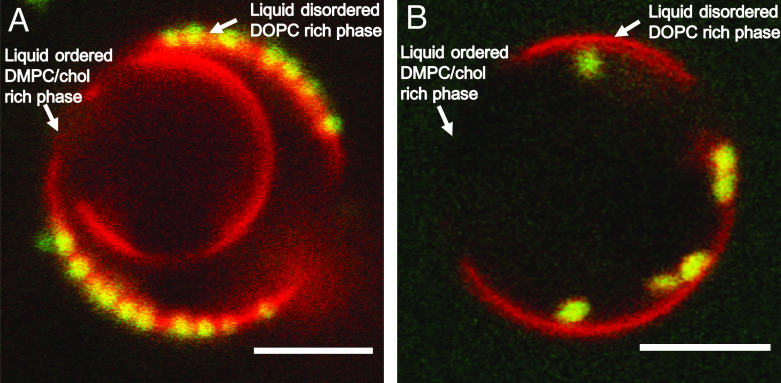
2D CLSM images of the adsorbed spherical MG1 microgels (*A*) and ellipsoidal MG2 microgels (b/a = 2) (*B*) on GUVs composed of DOPC, DMPC, and cholesterol (DOPC/DMPC/chol, molar ratio 7:7:3), forming coexisting liquid-ordered and liquid-disordered membranes phases. Temperature: 16 ^°^C. (Scale bars, 5 μm.)

First, we prepared GUVs from either of the lipid model systems mixed with the red fluorescent lipid analogue Rhod-PE (0.5 mol%). The green-labeled microgels were then added to the microfluidic channel, and the association of microgel particles with the GUVs was observed using CLSM. An overview of the findings is given in Fig. 2, showing that the microgel particles are either positioned at the membrane surface with the long axis parallel to the membrane plane or deeply wrapped by the lipid membrane with the long axis perpendicular to the vesicle surface. Time-resolved imaging of the step of the adsorption process shows that irrespectively of the orientation at which the ellipsoidal particles approach the membrane, they are always landing at the membrane with their long axis parallel to the membrane (Movies S1–S3 and *SI Appendix*, Fig. S6) before they, for some compositions, further become wrapped by the membrane. The images shown in Figs. 2–4 were all obtained approximately 2 h after the mixing of particles with the lipid vesicles when the system had reached a stable state both with respect to particle wrapping and water transport across the membrane. The GUVs are prepared in pure MilliQ water with no added solute or buffer, and after equilibration with respect to the water transport, the vesicles are assumed to have zero tension. The spherical MG1 microgels adsorb at the surface of the bilayer for all vesicle systems investigated (Fig. 2*A*–*C*). A closer inspection of the images shows that the DOPC and DMPC membranes are slightly deformed around the particles, here denoted as shallow wrapping, while for the DMPC/chol system, no membrane deformation can be distinguished (*SI Appendix*, Fig. S7). For the ellipsoidal microgel particles, the adsorption behavior is clearly different from that of the spherical ones. We study two types of ellipsoidal microgel, MG2 and MG3, with aspect ratios (b/a) 2 and 6, respectively. For some of the membrane compositions investigated, these ellipsoidal particles are almost completely engulfed by the membrane. This state is here denoted as deep wrapping. The general trend is that deep wrapping occurs for the lipid membranes with the lowest bending rigidity and largest effective lipid headgroup area (most hydrophobic membrane interface) and for the microgels with the largest aspect ratio (Fig. 2*F*, *H*, and *I* and *SI Appendix*, Fig. S8). The ellipsoidal MG2 microgels are adsorbed with the long axis parallel to the membrane for the lipid bilayers composed of DMPC and DMPC/chol (Fig. 2 *D* and *E*), while they are deeply wrapped for the bilayer composed of DOPC (Fig. 2*F*). For the MG3 microgel particles with the highest aspect ratio, deep wrapping occurs both for DOPC and DMPC membranes (Fig. 2 *H* and *I*), while the particles are shallow wrapped for the most rigid DMPC/chol membrane (Fig. 2*G*). Furthermore, it is noted that the lipid-wrapped microgels are in most cases not separated as isolated particles along the membrane but were rather found to aggregate at the inner side of the membrane.

Fig. 3 shows 3D-reconstructed images for the shallow-wrapped particles that are located at the membrane surface from Fig. 2*A*–*E*, and *G*. It is clear that the surface-adsorbed spherical MG1 and ellipsoidal MG2 microgels are closely packed with a nonrandom organization. The spherical microgels form 2D colloidal crystals with hexagonal structure for all membrane systems (Fig. 3*A*–*C*). The spherical microgels are less closely packed on the least rigid DOPC membrane, with average MG1 center-to-center distances measured as 1.20 ± 0.05 μm, as compared to the average distances of 1.07 ± 0.03 μm and 1.03 ± 0.02 μm on the DMPC and DMPC/chol membranes, respectively. The particle separation can be compared to the diameter of the spherical microgel that was determined to 0.92 μm by dynamic light scattering (DLS) (*SI Appendix*, Fig. S4). The hexagonal arrangement of the spherical MG1 microgels closely resembles previous observations for PNIPAM microgels on fluid DMPC and DOPC membranes (45, 46). For the shallow-wrapped ellipsoidal MG2 microgel, we observe regions with local side-to-side “smectic-like” ordering at liquid-disordered DMPC membranes (Fig. 3*E*). In case of the more rigid liquid-ordered DMPC/chol membrane, the ellipsoidal microgels distribute more uniformly at the membrane surface, with some hexagonal positional ordering but without any clear orientational correlation between the particles reminiscent of a “plastic crystal”-like configuration (Fig. 3*D*). Finally, the ellipsoidal MG3 microgels with the highest aspect ratio instead show random distribution and orientation at the membrane surface (Fig. 3*F*). We also investigated the spherical and ellipsoidal microgels adsorbing to DOPC GUVs at a lower temperature of 20 ^°^C (*SI Appendix*, Figs. S9 and S10), showing very similar behavior as the same systems at the higher temperatures (Figs. 2*C*, *F*, and *I* and 3*C*).

### Preferential Adsorption at the Liquid-Disordered Bilayer.

We next study the competition in microgel adsorption between segregated domains in the membrane with different membrane rigidities and acyl-chain order. For this purpose, we used GUVs composed of segregated domains formed by DOPC-rich liquid-disordered and DMPC/chol-rich liquid-ordered bilayer phases (*SI Appendix*, Fig. S11) (77, 78). Here, the red fluorescent lipid analogue Rh-PE preferentially locates in the more disordered DOPC-rich phase and is depleted from the liquid-ordered phase, which appear black in the confocal images. The spherical MG1 and the ellipsoidal MG2 microgels were then added to the GUVs with segregated phases (Fig. 4 *A* and *B*). Both, spherical and the ellipsoidal microgels strongly prefer the less rigid DOPC-rich liquid-disordered phase. In line with the findings for the single-phase DOPC vesicles (Fig. 2 *C* and *F*), the spherical particles are only shallow-wrapped by the membrane and positioned at the vesicles surface, while the ellipsoidal particles are deeply wrapped by the membrane. It is also noted that for the present conditions when microgels are not present in excess, there is no observable microgel adsorption on the liquid-ordered DMPC/chol-rich domains (dark).

### Wrapping Energies and Prediction of Wrapping States.

We performed a detailed numerical analysis of the energies that arise from the change of membrane curvature and the variation of microgel-membrane contact area during the wrapping process. The calculations are based on a continuum model for the membrane with curvature elasticity and lateral tension. The total energy of a particle attached to a lipid bilayer is the sum of membrane deformation energy, integrated over the entire membrane area, and microgel-membrane adhesion energy (17, 31, 79), i.e.,

[1] $$\begin{array}{c} E=\;\int_{A}dS\;\left[2\kappa \;H^{2}+\sigma \;\right]-\int_{A_{\;\text{ad}}}dS\;w, \end{array}$$

Here, *A* is the total area of the membrane and *A*_ad_ the area of the membrane that is adhered to the particle. The local membrane shape is described by the mean curvature *H* = (*c*_1_ + *c*_2_)/2, where *c*_1_ and *c*_2_ are the principal curvatures. The curvature-elastic properties of the membrane are characterized by the bending rigidity *κ* and the lateral tension *σ*; the interaction between microgel and lipid bilayer is modeled as contact interaction with adhesion strength *w*.

Membrane shapes as well as deformation and adhesion energies were calculated using triangulated membranes and energy minimization with the help of “Surface Evolver” (80, 81). Importantly, we include not only the deformation of the adhered, but also of the free, nonadhered part of the membrane, which is numerically challenging for nonsymmetric conformations.

**Fig. 5.**
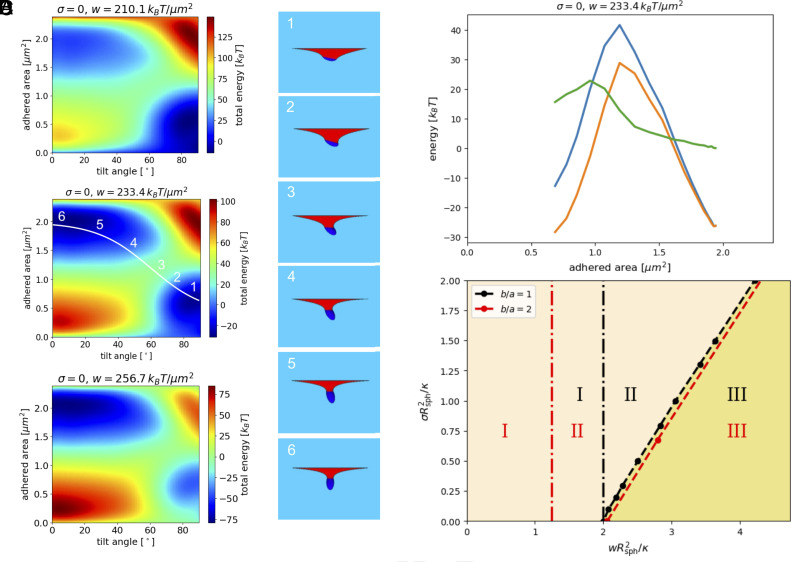
Wrapping energies for ellipsoidal particles with aspect ratio *b*/*a* = 2 and volume *V*_0_ = 0.31 μm^3^ at tensionless and initially planar lipid-bilayer membranes with bending rigidity *κ* = 20 *k*_*B*_*T*. (*A*) Wrapping energy landscapes as a function of the ellipsoids orientation for *w* = 210.1, 233.4, and 256.7 *k*_*B*_*T*/μm^2^. The transition between the submarine and the rocket states with adhesion strength *w* = 233.4 *k*_*B*_*T*/μm^2^ illustrated by simulation snapshots along the transition path shown in (*B*). (*C*) Energy along the transition path *A*_*ad*_ = 0.8(1.5 − tanh(0.03(*θ* − 60 ° )) μm^2^: total energy (blue), energy of attached membrane (orange), and energy of the free membrane (green). (*D*) Wrapping of particles with fixed volume *V*_0_ = 4/3*πR*_sph_^3^ at initially planar lipid membranes with bending rigidity *κ* for various tensions *σ*. The vertical dashed-dotted lines indicate the transition from the nonwrapped to the shallow-wrapped state. Phases for an ellipsoidal particle with aspect ratio *b*/*a* = 2 (red lines) and for a spherical particle (black lines) for various membrane tensions *σ* and adhesion strengths *w*. I: nonwrapped; II: shallow-wrapped; III: deep-/complete-wrapped.

From these calculations, wrapping fractions and orientation angles of microgels at an initially planar lipid-bilayer membrane can be predicted. All particles are rotational ellipsoids with the same fixed volume *V*_0_ and minor and major semiaxes *a* and *b*, respectively. Fig. 5*A* shows the wrapping-energy landscapes for an ellipsoidal particle with aspect ratio *b*/*a* = 2 as a function of the adhered membrane area *A*_ad_ and the orientation angle *θ* of the long axis with respect to the membrane normal for different *w*-values. For low adhesion strengths, we find a shallow-wrapped state with “submarine” orientation, where the long axis is oriented parallel to the membrane surface which avoids the wrapping of the highly curved tips of the particles. For high adhesion strengths, we find a deep-wrapped state with “rocket” orientation, where the long axis is oriented perpendicular to the membrane, and one of the curved tips is wrapped.

In Fig. 5*B*, the transition path between the “submarine” and “rocket” states is illustrated by simulation snapshots at intermediate adhesion strengths. At coexistence, the shallow-wrapped and deep-wrapped states are separated by an energy barrier corresponding to the energy needed for wrapping of the highly curved tip of the particle (Fig. 5*C*). The energy barrier is obtained as the energy difference along the optimal transition path between the energies at the saddle in the energy landscape and of the two stable states. It is important to note that this energy barrier for elongated particles depends on the tilt angle, which is varying smoothly from 90 ^°^ (submarine) to 0 ^°^ (rocket) along the transition path.

For a membrane under lateral tension, the adhesion strengths for the transition between the shallow-wrapped and the deep-wrapped state of both spherical and ellipsoidal particles increase linearly with increasing tension; see Fig. 5*D*. Membrane conformations for tilted ellipsoidal as well as spherical particles with various tensions are shown in *SI Appendix*, Fig. S13. Interestingly, for aspect ratio *b*/*a* = 2, the transition from the shallow-wrapped to the deep-wrapped state for ellipsoidal particles occurs at almost the same adhesion strengths as for spherical particles. The hindrance of particle wrapping, and promotion of shallow-wrapped states with increasing tension, occurs because the extra membrane area needed for wrapping has to be pulled in against the lateral tension (17, 31). The energy barrier between shallow- and deep-wrapped states increases with increasing membrane tension—an effect which becomes more pronounced with increasing aspect ratio (*SI Appendix*, Fig. S12).

**Fig. 6.**
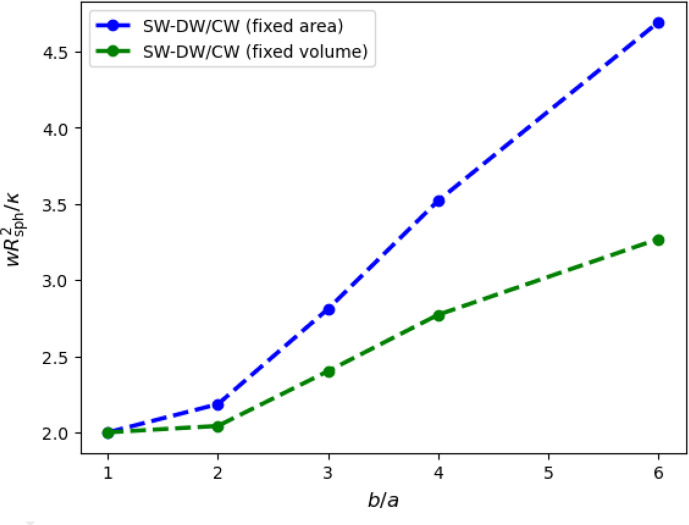
Scaled adhesion strength at the wrapping transition for ellipsoidal particles with aspect ratios 1 ≤ *b*/*a* ≤ 6 at tensionless and initially planar lipid-bilayer membranes for fixed particle surface area *S*_0_ and *R*_sph_ = (*S*_0_/4*π*)^1/2^, and fixed particle volume *V*_0_ and *R*_sph_ = (3*V*_0_/4*π*)^1/3^.

The calculated dependence of the adhesion strength at the wrapping transition on the aspect ratio *b*/*a* is shown in Fig. 6, over the same range of aspect ratios as studied in the experiments. The figure illustrates that beyond *b*/*a* = 2, the adhesion strength rapidly increases with aspect ratio—in contrast to the experimental observations. This will be discussed in detail below. Furthermore, in the comparison of our calculations, which take into account the deformation of the nonadhered membrane, the transition values of *w* in the range 2 ≤ *b*/*a* ≤ 6 agree very well with those of an approximation with a planar nonadhered membrane (82, 83). The reason for this somewhat-surprising agreement must be that the neck-like membrane deformation is close to catenoidal and therefore is not associated with any significant curvature-energy cost.

A simple analytical approximation for the phase boundary can be obtained by considering the coexistence of a nonwrapped and a complete-wrapped state (in contrast to shallow- and deep-wrapped states). This provides the linear dependence *w* = *σ* + *F*_*b*_(*b*/*a*)/*A*_0_ because both surface-tension and adhesion energy contributions are proportional to the particle surface area *A*_0_. Here, *F*_*b*_ is the bending energy for the complete wrapping of the particle, which—because of the scale invariance of the curvature energy—depends only on the aspect ratio, but not on the particle size. This linear relation of *σ* and *w* at the transition agrees well with our numerical result for both spherical and ellipsoidal particles; see Fig. 5*D*. Because the deep-wrapped state of the ellipsoidal particle leaves one highly curved tip unwrapped, the ellipsoidal particles with aspect rate *b*/*a* = 2 are wrapped at basically the same adhesion strengths than spherical particles, independent of the lateral membrane tension.

For larger aspect ratios *b*/*a* > 2, complete wrapping of ellipsoidal particles becomes increasingly unfavorable with increasing *b*/*a* (at fixed volume) because of the increasing surface curvature; see Fig. 6 and *SI Appendix*, Fig. S14. This can be seen most easily analytically for isochoric spherocylinders, for which *F*_*b*_ = 4*πκb*/*a* + 4*π*, which at constant volume *V*_0_ is 4*πV*_0_/*a*^3^ + 4*π* for high aspect ratios. Thus, the adhesion strength for the wrapping transition is expected to increase linearly with *b*/*a* in this approximation. Possible ways to reduce the particularly high wrapping energy cost not only at the “free” tip in the deep-wrapped (but not complete-wrapped) state but also at the “buried” tip include the formation of a hole or a blister at the tip, as discussed in *SI Appendix*. This can lead to a reduction of the energy cost for the formation of deep-wrapped states for ellipsoids of high aspect ratio; see *SI Appendix*, Figs. S15–S19.

## Discussion

The experimental data clearly demonstrate that basic physical properties, including particle shape, membrane rigidity, and effective lipid headgroup area in the membrane (apparent hydrophobicity), can be used to direct the adsorption and wrapping of microgel particles at lipid membranes. The experiments show that the deep-wrapped states are more favorable for the membranes with the lower bending rigidity and higher effective headgroup area and for ellipsoidal microgel particles with high aspect ratios compared to spherical particles of similar volume. Shallow-wrapped ellipsoidal particles are always oriented with their long axis parallel to the membrane, deep-wrapped particles often with the long axis perpendicular to the membrane (Figs. 2 and 3), as also predicted theoretically for similar systems (32). The time-resolved experiments (*SI Appendix*, Fig. S6 and Movies S1–S3) further show that even when the ellipsoidal microgels adsorb in a different angle, they reorient when adsorbed to maximize the interfacial contact area. Taken together, this implies strong attractive interactions between the microgels and the lipid membranes. Furthermore, the packing of the lipid chains clearly impacts the particle adsorption. The adsorption is much higher to the liquid-disordered membrane with less closely packed acyl-chains as compared to the liquid-ordered membrane, as inferred from the observation of nonuniform distribution of particles to a segregated membrane with domains composed of these different phases (Fig. 4).

In colloidal systems, nonspecific attractive forces typically originate from either hydrophobic or electrostatic interactions (84). In the present system, the microgel particles are slightly positively charged (*SI Appendix*, Table S1), while all three lipid model systems are composed of zwitterionic PC lipids that have an overall zero charge at neutral solution conditions. It is here important to emphasize that the membrane physical properties were varied by varying the acyl-chain composition, while the lipid headgroup chemistry is the same for all model membranes investigated. If the adsorption of particles would be dominated by electrostatic attraction between the cationic particles and the zwitterionic membrane interface, one would expect similar particle adsorption to both types of domains in Fig. 4, which is not the case. All experiments were performed at temperatures ranging between 16 and 28 ^°^C, which is clearly below the volume phase transition temperature (VPTT) of PNIPMAM microgel (42 ^°^C) (61). We therefore expect the PNIPMAM polymer chains of the microgel particles to be extended and flexible. The difference in lipid packing between liquid-disordered and liquid-ordered phases implies differences in the exposure of the hydrophobic hydrocarbon chains (85). We therefore suggest that the strong attraction between the microgel and the PC lipid membranes is mainly attributed to partial partitioning of the protruding dangling polymer chains into the hydrophobic lipid chains exposed at the membrane interface. One can expect particle–membrane adhesion energy to be higher for the DOPC membrane as compared to the other model membranes investigated due to the larger effective headgroup area and thus larger exposure to the hydrophobic membrane interior (85) may in turn lead to a stronger drive for membrane wrapping. This is also in line with the proposed membrane adhesion caused by partitioning of hydrophobic residues of amphipathic peptides or hydrophobic bases of single-stranded RNA into lipid packing defects in fluid membranes (86, 87).

The modeling of the wrapping of ellipsoidal particles by membranes provides the energies for various orientations and wrapping fractions for both tense membrane and membranes with zero tension, going beyond analytical bending-energy-only calculations (83). In comparison to former studies (31, 32), our theoretical calculations reassess the effect of shape anisotropy on membrane wrapping by accounting for the free-membrane contribution and membrane tension for all tilt angles. Our calculations show that the transition to the deep-wrapped state occurs at similar adhesion strength for spherical and shorter ellipsoidal (*b*/*a* = 2) particles and that longer ellipsoids are more difficult to wrap than shorter ellipsoids or spheres. The comparison between the experimental and theoretical data implies that small changes in particle surface properties upon stretching, which can cause an increased membrane adhesiveness (*SI Appendix*, Figs. S1–S5 and Table S1), are required to rationalize the experimental observations. Additionally, pore or blister formation at the buried particle tip may reduce the energetic cost of wrapping more elongated ellipsoids, as discussed in *SI Appendix*, Figs. S15–S19. However, their contribution alone is not sufficient to support the experimental observations and the preferential wrapping of highly elongated microgels (*SI Appendix*, Fig. S16). We here also point out that the absence of visible undulations in the experiments is not by itself a sign of tense vesicles. According to theoretical predictions (88), quasi-spherical tensionless vesicles would have the fluctuation amplitudes < |*u*_*l*, *m*_|^2^ > = *k*_*B*_*T*/[*κ* ⋅ *l*(*l* − 1)(*l* + 1)(*l* + 2)] (in units of the vesicle radius) in an expansion of the vesicle shape in spherical harmonics of mode numbers *l*,*m*. For *κ*/*k*_*B*_*T* = 50, the dominant ellipsoidal deformation (with *l* = 2) is thus expected to have an amplitude $<|u_{2,m}{|}^{2}{>}^{\frac{1}{2}}R=R/35=150$ nm for a vesicle radius *R* = 5 μm, which would not be observable in the present experiments

It is here important to note that particle softness was not considered in the simulations. As the microgel shells are compressible, the uniaxial stretching process may not only lead to a reduction of volume but also to a reduced swelling of the microgel shell. The consecutive densification of the microgel shell and decrease in microgel softness with increasing aspect ratio may thus alter the adhesion energy to the membrane. These effects may contribute to preferential wrapping at the membrane as previously discussed (89, 90). However, more detailed models and simulations still need to be developed to account for the microgel structure and bulk elasticity and its repercussions on the wrapping behavior.

Membrane-mediated interactions play an important role in the microgel organization at the membrane (45). The spherical microgels arrange into a highly ordered and closely packed 2D crystalline structure at the fluid membrane, which implies high lateral diffusion of particles in the plane of the bilayer. Membrane-mediated repulsive interactions induced by partial wrapping of the particles are here characterized by an average intermicrogel distance that is larger than the hydrodynamic diameter of the particles. By increasing *κ*, and thus reducing the degree of wrapping, the intermicrogel distance further decreases. Taken together, this indicates the possibility to control the microgel spacing by mediating the membrane properties and particle–membrane interactions. For the ellipsoidal microgels, we also observed dense packing of the shallow-wrapped MG2 ellipsoids with local side-to-side association at the DMPC liquid-disordered membrane and hexagonal arrangements reassembling plastic crystal characterized by a well-defined positional ordering and no orientational correlation at the liquid-ordered DMPC/cholesterol membrane. Interestingly, in this case, the average interparticle distance increases with increasing *κ* and decreasing effective headgroup area, conversely to the spherical MG1 microgels as the interactions of shallow-wrapped ellipsoids become less directional. Finally, the highly elongated MG3 only sparsely adsorbed at the GUVs. This observation is surprising as one could expect that for such a high aspect ratio, a clear side-to-side or at least nematic assembly may be observed. The observed effects may be related to the reduced mobility of such ellipsoids at the membrane interface or to the difference of curvature with the GUVs. Our experiments highlight the possibility offered by the combination of shape anisotropy and membrane-mediated interactions to open up for various self-assembly structures that differ, for instance, from assembly mediated by capillary interactions (91–94). Hereby, the capillary assembly of soft anisotropic core–shell (92–94) and hollow (94) microgels offers even more possibilities as the extent of both lateral and longitudinal deformations is strongly influenced by the microgel design. The same is expected to hold for the membrane-mediated interactions, for which different anisotropic microgel designs may result in a rich assembly behavior. The wrapping behavior may further be tuned by alteration in membrane tension, osmotic imbalance, and temperature-induced changes in membrane assembly and microgel configuration.

## Materials and Methods

### Materials.

*N*-isopropylmethacrylamide (NIPMAM; Sigma-Aldrich), *N*,*N*′-methylenebisacrylamide (BIS; Fluka), hexadecyltrimethylammonium bromide (CTAB; Sigma-Aldrich), and 2,2′-Azobis(2-methylpropionamidine) dihydrochloride (V50; Fluka) were used as received. Styrene monomer (BASF) was purified on an Al_2_O_3_ column prior to use. The following were purchased from Avanti Polar Lipids: 1,2-dioleoyl-sn-glycero-3-phosphocholine (DOPC, C_44_H_84_NO_8*P*_), 1,2-ditetradecanoyl-sn-glycero-3-phosphocholine (DMPC, C_36_H_72_NO_8*P*_), and 1,2-dioleoyl-sn-glycero-3-phosphoethanolamine-N-(lissamine rhodamine B sulfonyl) (ammonium salt) (Liss Rhod PE, C_68_H_109_N_4_O_14_PS_2_). Alexa Fluor^TM^ 488 NHS Ester (C_25_H_15_Li_2_N_3_O_13_S_2_) was purchased from Thermo Fisher Scientific Inc. Cholesterol (C_27_H_46_O), chloroform (analytical grade, ≥ 99.8%), methanol (analytical grade, ≥ 99.9%), Mini Dialysis Kit (molecular weight cutoff, 8 kDa; GE Healthcare) were purchased from Sigma-Aldrich. MilliQ water was used in all the experiments.

### Microgel Synthesis and Characterization.

#### Core synthesis.

Polystyrene core particles were first synthesized by emulsion polymerization and then employed as seeds for radical polymerization of the cross-linked shell. Emulsion polymerization was performed in a 1-L three-necked round-bottom flask equipped with a stirrer, a reflux condenser, and a thermometer. Under continuous magnetic stirring, 0.014 g CTAB and 2.625 g NIPMAM were dissolved in 50 mL MilliQ water. After the addition of 48.3 g styrene, the mixture was degassed with nitrogen for 20 min and then heated to 80 ^°^C under a nitrogen atmosphere. In 9.2 mL water, 90 mg of the V50 initiator was dissolved. The degassed initiator solution was added dropwise while the mixture was stirred at 300 rpm. The reaction was carried out for 8 h under a nitrogen atmosphere. The latex dispersion was then cooled down to room temperature and filtered through glass wool to remove traces of coagulum. Purification was performed by dialysis of the latex dispersion against pure MilliQ water solution for 3 wk (Medicell, 12,000 to 14,000 Da). At the end of the process, a 20.2 wt% core stock dispersion was obtained. We notice that without the addition of a small amount of NIPMAM, no core–shell can be obtained in the second reaction step. This addition ensures that the surface of the core becomes hydrophobic at high temperatures, which enables the adsorption of PNIPMAM to the core during the core–shell synthesis.

### Core–Shell Microgels Synthesis.

The seeded emulsion polymerization for the core–shell system was carried out using 32 g of our stock core solution (5.1 wt%). In 10 mL water, 2 mg fluorescein-5-isothiocyanate (FITC) was dissolved. To 57 g water and to the core solution, 1.61 g NIPMAM, 0.1 g BIS, and the FITC solution were added. The mixture was degassed with nitrogen for 20 min and then heated to 80 ^°^C. One hour later, the reaction was started by the slow addition of 50 mg V50 (dissolved in 5 mL of water), and the entire mixture was stirred at 300 rpm for 8 h at 80 ^°^C under a nitrogen atmosphere. After cooling to room temperature, the core–shell latex dispersion was filtered through glass wool and purified by dialysis against MilliQ water for 2 wk. This spherical core–shell microgel will be referred to as MG1.

### Postprocessing of the Core–Shell Microgels into Prolates.

The anisotropic particles were obtained using the mechanical stretching method for PS lattices first proposed by Ho et al. (95) and adapted to core–shell microgels as described in our former studies (62, 65). In a nutshell, the core–shell particles were embedded into a PVA film. Film stripes were then clamped in a custom-made automatized uniaxial deformation set up in a silicon oil bath maintained at 145 ^°^C. The stripes were stretched at different draw ratios corresponding to deformations *γ* = *ΔL*/*L*_0_ (where *Δ* L is the difference in length before and after stretching and *L*_0_ the initial length of the film) of 50, and 400%, cooled down to room temperature and sorted according to their local deformation *γ*, accessible via a grid initially sketched at the surface of the film. The cleansing procedure to recover the stretched particles is described in our former studies (62, 65). Stretching the particles above the *T*_*g*_ of the polystyrene core ensures that the particles deform plastically, which in turn results in the postprocessing of the initially spherical core–shell microgels into ellipsoidal core–shell microgels. The anisotropic particles with deformation of 50% and 400% will be referred to as MG2 and MG3, respectively. In order to enhance the fluorescence signal, the microgels were fluorescently labeled with Alexa Fluor 488 N-hydroxysuccinimidyl ester (Alexa Fluor^TM^ 488 NHS Ester, C_25_H_15_Li_2_N_3_O_13_S_2_) and followed by overnight dialysis against water to remove excess dye.

### Core–Shell Microgel Characterization.

The microgels were characterized by transmission electron microscopy (TEM) (*SI Appendix*, Figs. S1 and S2), CLSM (*SI Appendix*, Fig. S3), DLS and electrophoretic mobility measurements (*SI Appendix*, Fig. S4). The measurements were performed at 20 and 28 ^°^C. The results are summarized in *SI Appendix*, Table S1. The mean aspect ratio (b/a) of the nonspherical microgels MG2 and MG3 was determined at 28 ^°^C from statistical analysis over 100 microgels adsorbed at the cover glass in water, respectively. The three microgels are slightly positively charged. The microgels were also observed at superresolution using a 3D-structured illumination microscope (N-SIM, Nikon Healthcare) mounted on a Nikon Ti2 research microscope body equipped with an SR HP apochromat TIRF 100x/NA 1.49 oil-immersion objective lens and a Hamamatsu ORCA-Flash4.0 V3 CMOS camera. SIM images of the Alexa488-stained microgels were acquired at 20 ^°^C with 700-ms exposure time and no binning and reconstructed using the N-SIM module in the NIS-Elements AR software package. Illumination modulation contrast, high-resolution noise suppression, and out-of-focus blur suppression were set to 2.50, 1.0, and 0.35, respectively. Finally, the images presented in this manuscript were adjusted for brightness and contrast (*SI Appendix*, Fig. S3).

### GUV Preparation.

GUVs were prepared on indium tin oxide (ITO)-coated coverslips (30 to 60 Ohms/Sq, Sigma-Aldrich) by the electroformation method in a microfluidic channel (45, 96); the setup used in the study is same as in our previous work (45). Stock solutions of either DOPC, DMPC, and DMPC with 30 mol% cholesterol (DMPC/chol) or DOPC and DMPC with cholesterol (DOPC/DMPC/chol, molar ratio 7:7:3) were prepared in chloroform/methanol (9:1 volume ratio) at a concentration of 0.2 mg/mL. To all lipid samples, 0.5 mol% the fluorescent lipid analogue Liss Rhod PE (red) was added. To prepare GUVs, the ITO-coated coverslips were first cleaned with ethanol and dried by nitrogen gas. Then, 10μL lipid solution was deposited onto the conductive side of the ITO-coated coverslip and dried in a vacuum chamber for overnight. The lipid-coated coverslip was then mounted to a self-adhesive underside of a microchannel (Ibidi sticky-Slide VI 0.4). Another ITO-coated coverslip was attached to the top side of the microchannel with the conductive side toward the sample solution. Next, conductive wires were used to connect the conductive sides of the two ITO-coated coverslips to the electrodes from the frequency generator. The AC-electric field (10 Hz, 3V) was applied for 3 h to generate the GUVs. The GUVs were prepared in the fluid state, DOPC at 20 or 28 ^°^C and DMPC, DMPC/chol, and DOPC/DMPC/chol at 28 ^°^C.

### Confocal Laser Scanning Microscope.

The fluorescent GUV/microgel samples were observed by CLSM (Leica SP5) operated in the inverted mode (D6000I). The temperature of the samples was control with an accuracy of 0.2 ^°^C by mounting the CLSM to a thermostated enclosure. Samples were equilibrated for 2 h at 20 or 28 ^°^C before observation. The red fluorescence of the membrane lipid analogue (Liss Rhod PE) and the green fluorescence probe associated to the microgel particles, Alexa Fluor^TM^ 488 NHS Ester, were excited by using a HeNe laser at 543 nm and an argon-ion laser at 488 nm, respectively. The 3D CLSM images were reconstructed from confocal z-stack images.

Dynamics of MG2 particle adsorption to lipid membranes were acquired using a Nikon A1 confocal Ti-2 microscope with NIS-Elements AR software version 5.20.02. Alexa 488 (microgels) and Rhodamine (vesicles) were excited simultaneously using a 488-nm and 561-nm laser line, respectively. Fluorescence emission from 500 to 550 nm (Atto 488) and 570 to 620 nm (Rhodamine) was collected in parallel with a Nikon DU4 4-channel detector. A Plan Apo *λ* 100x Oil/1.45 objective was used with the pinhole set to 48 μm. Frames were recorded with two times line averaging to build z-stacks with a 1-μm axial step size and a z-range of 16 μm, covering the height of the imaged vesicles. Several regions in the sample were imaged in the same sequence over a period of 20 min with each position being imaged every 11.9 s. Finally, the images were deconvolved via 15 iterations of Richardson–Lucy’s method in the NIS-Elements AR software.

### Experimental Procedure.

First, GUVs composed of DOPC, DMPC, or DMPC/chol were prepared by the electroformation method. After completing the preparation, the prepared GUVs were first observed by CLSM. Next, the microgels were added to the microchannel with the prepared GUVs. After particle adsorption equilibrium has been reached (around 2 h after adding microgels), the samples were observed by using CLSM. The preparation of GUVs and CLSM observation were carried out at 20 or 28 ^°^C. At the investigated temperature, DOPC and DMPC GUVs are in liquid-disordered phase, and DMPC/chol GUV is in liquid-ordered phase (97). For DOPC/DMPC/chol (molar ratio 7:7:3), the GUVs were prepared at 28 ^°^C, and CLSM observations were performed at 28 ^°^C first and then lowered to 16 ^°^C. The spherical MG1 or the ellipsoidal MG2 particles were added to the cell and followed by observing the interactions between the GUV and the microgels by CLSM at 16 ^°^C.

## Supplementary Material

Appendix 01 (PDF)

Movie S1. The adsorption and rotation of *MG2* microgels at DOPC GUVs was followed over time.

Movie S2. The adsorption and rotation of *MG2* microgels at DOPC GUVs was followed over time.

Movie S3. The adsorption and rotation of *MG2* microgels at DOPC GUVs was followed over time.

## Data Availability

All study data are included in the article and/or supporting information.
