# Supplementary material for: Wrapping anisotropic microgel particles in lipid membranes: Effects of particle shape and membrane rigidity
Source: Proc Natl Acad Sci U S A. 2023 Jul 17;120(30):e2217534120. doi: 10.1073/pnas.2217534120 (PMC10372639; doi:10.1073/pnas.2217534120)
Supplement: Supplementary file 1 — Appendix 01 (PDF) [file pnas.2217534120.sapp.pdf]

PNAS
www.pnas.org

Supplementary Information for

Wrapping anisotropic microgel particles in lipid membranes - effects of particle shape and membrane rigidity

Xiaoyan Liu, Thorsten Auth, Nabanita Hazra, Morten Frendø Ebbesen, Jonathan Brewer, Gerhard Gompper, Jérôme J. Crassous and Emma Sparr

Corresponding Author: Xiaoyan Liu
E-mail: xiaoyanl@kth.se

This PDF file includes:

Supplementary text
Figs. S1 to S19
Tables S1 to S2
Legends for Movies S1 to S3
SI References

Other supplementary materials for this manuscript include the following:

Movies S1 to S3

Supporting Information Text

Characterization of spherical and ellipsoidal microgel particles

Transmission electron microscopy (TEM). The samples were prepared by dropcasting a 1 wt% solution on a 300 mesh carbon-coated copper grid placed on a paper filter at room temperature. The micrographs were recorded on a TEM-CM100 (Philips) operating at an acceleration voltage of 80 keV. Figure S1 presents representative micrographs of the spherical core and core-shell particles (Fig. S1A-D) and the ellipsoidal core-shell microgels with increasing deformation (Fig. S1E-H). The core particles have average diameter of 429 ± 25 nm and the core-shell of 494 ± 31 nm. The elongated particles present a prolate shape and an increasing aspect ratio with increasing deformation as summarized in Fig. S2. The diameter of the collapsed spherical particles, the full long axis b_c and short axis a_c as well as the aspect ratio b_c/a_c of the collapsed ellipsoidal microgels were determined from statistical analysis of more than 100 particles. The micrographs confirm a low polydispersity in terms of size and shape in the order of 6-7% for b_c, a_c and b_c/a_c for $\gamma = 50\%$. The most anisotropic particles are less defined particularly in terms of their length, with polydispersity for b_c/a_c in the order of 14 %. The different results were compared to uniform deformation conditions considering an isochore deformation, where $b_c = R_c(1+\gamma)$, $a_c = R_c(1+\gamma)^{-0.5}$ and $b_c/a_c = (1+\gamma)^{1.5}$, where R_c refers to the radius of the original spherical particles. We can observe that the particles deform more at low γ than expected for a uniform deformation similarly to our former observation on anionic core-shell microgels synthesized with KPS instead of V50 (1). Remarkably, the variation of b_c/a_c measured for the present cationic microgels as function of γ is almost identical to the reported values for the anionic core-shell microgels confirming the robustness of the post-processing into prolates. It is also worthwhile noting that the most elongated microgels still preserve their ellipsoidal shape, whereas the "larger" anionic particles were significantly bending at a similar 400% deformation (1).

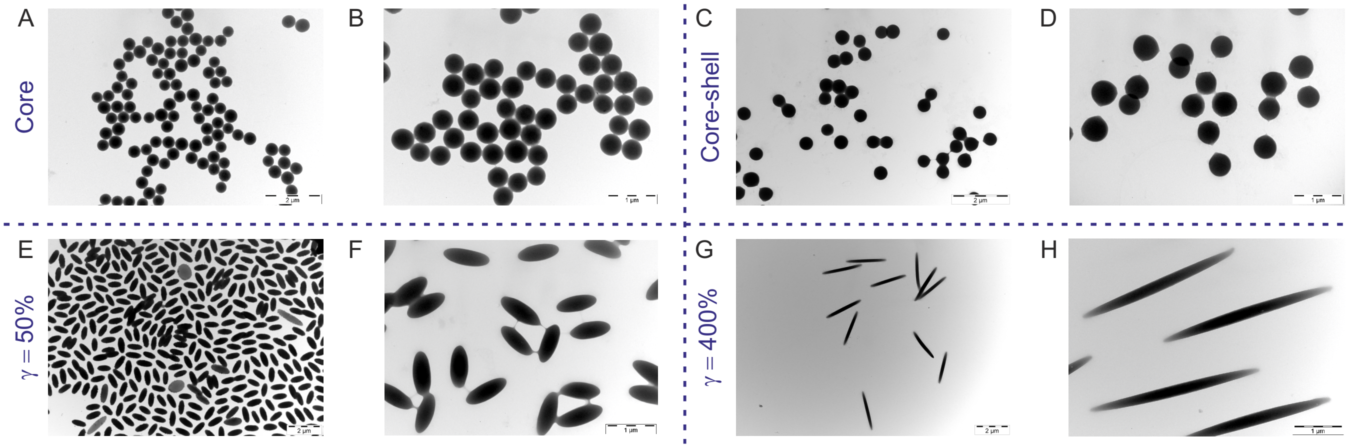

Fig. S1. TEM micrographs of the spherical core (A,B) and core-shell (C,D) particles. (E-H) Micrographs of the ellipsoidal core-shell microgels post-processed at deformations γ = 50% (E,F) and 400% (G-H), respectively.

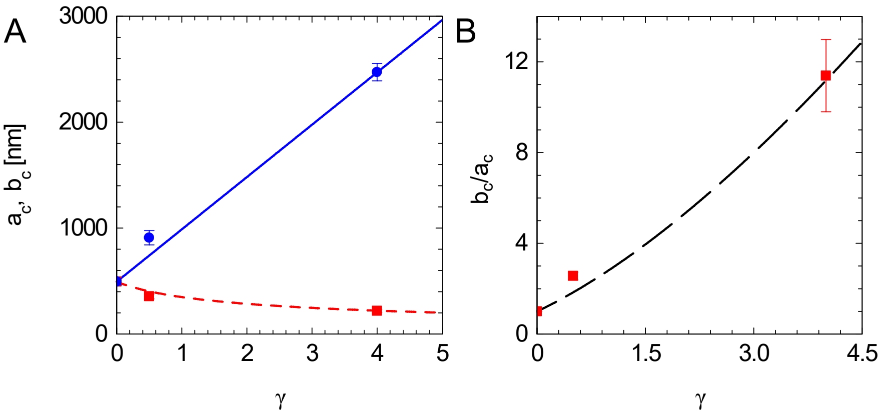

Fig. S2. (A) Evaluation of the long axis (b_c, circles) and short axis (a_c, squares) from statistical analysis of TEM micrographs as function of the film deformation γ. (B) Dependence of the aspect ratio b_c/a_c on γ. The lines represent the expected scaling for a uniform deformation.

Confocal laser scanning microscopy (CLSM) and super resolution characterization. The microgels labeled with Alexa488 used for the association with GUVs were imaged in their swollen configuration when adsorbed at the surface of the cover glass

at 20 °C. Typical CLSM micrographs are shown in Fig. S3 (top). The latter were statistically evaluated to estimate their average dimensions, b_s and a_s, and aspect ratio b_s/a_s. As expected, the dimensions are larger than from the TEM analysis due to the swelling of the microgel shell in water. It results in a decrease of their aspect ratio as summarized in Table S1. In addition, super-resolution microscope 3D-structured illumination microscopy (SIM) was performed on the same systems as shown in Fig. S3 (bottom). Such technique allow to clearly distinguish the core and the shell of the particles. Keeping in mind that an affine deformation into prolate of the core-shell particles would imply that the shell in the direction of the short axis Δa_s gets thinner, while the shell in the direction of the long axis Δb_s should become thicker such that $\Delta b_s/\Delta a_s \approx b_s/a_s$. However, the shell thickness remains relatively thin at the tips compared to the sides of the particles particularly for $MG3$, for which $\Delta b_s/\Delta a_s \approx 2.3$, whereas $b_s/a_s \approx 6$. The PNIPMAm shell is indeed not covalently attached to the core particle, we therefore conclude that the shell get highly compressed at the tips during the uniaxial stretching process. This is visible from the non-covalent labeling with Alexa 488 which is negatively charged. Indeed the microgel fluorescence is significantly brighter at the tip of the prolate and this effect is getting more pronounced at higher γ. The transformation into prolates may therefore not only have consequences on the conformation of the microgels and therefore its swelling and mechanical properties, but also on the distribution of charges and local adhesive properties of the ellipsoidal microgels.

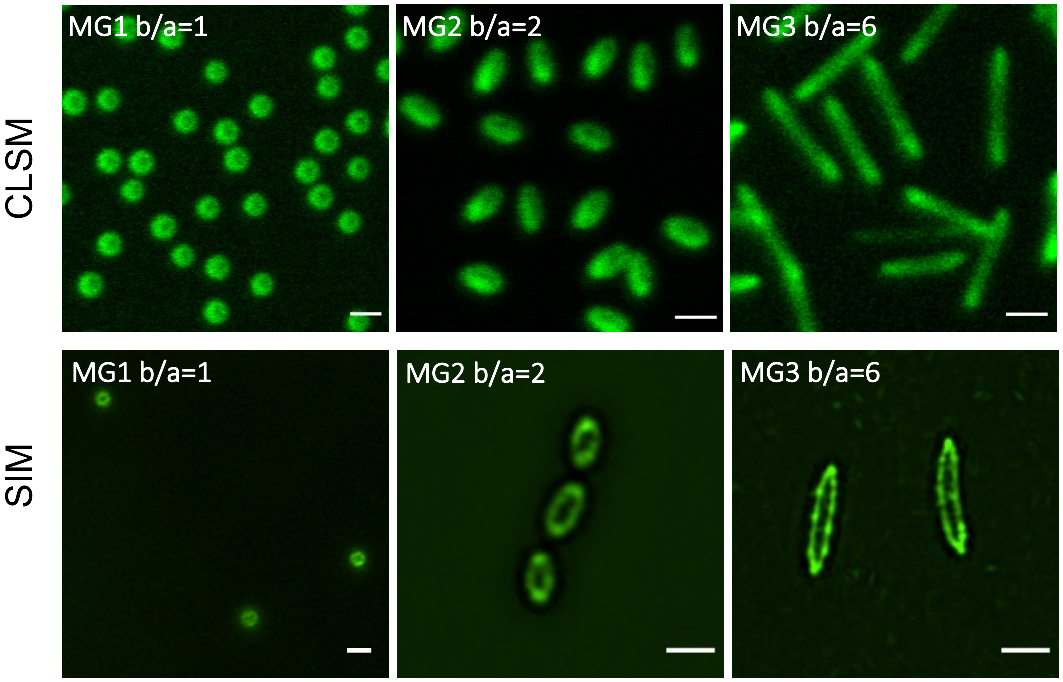

Fig. S3. (Top) 2D CLSM micrographs of the microgels obtained for different deformations γ adsorbed at the glass coverslip in their swollen configuration at 20 °C. (Bottom) Same microgel measured with super resolution microscopy SIM at 20 °C. Scale bars: 1 µm.

Dynamic light scattering (DLS). The diffusion of the particles was determined using dynamic light scattering. Dilute suspensions (0.001 wt%) were measured on an ALV setup at 20 and 28°C at low scattering angles ranging from 30 to 50° with 5° step. The translational diffusion coefficient D_T was determined from the linear dependence of the decay rate Γ versus the square of the scattering vector q^2 ($\Gamma = Dq^2$) derived from a first order cumulant analysis as shown in Fig. S4A,B. The hydrodynamic radius R_H of the spherical particles was determined from D_T via the Stockes–Einstein relation, $D_T = k_B T/6\pi\eta R_H$, with k_B the Boltzmann constant, T the temperature of the measurement in Kelvin and η the viscosity in Pa.s. For the core-shell ellipsoids the friction coefficient, f_p, is dependent on the dimensions and anisotropy of the prolates as follows (2, 3):

$$f_p = \frac{3\pi\eta\ b_H \sqrt{(b_s/a_s)^2 - 1}}{(b_s/a_s) ln\left((b_s/a_s) + \sqrt{(b_s/a_s)^2 - 1}\right)} \qquad [1]$$

D_T is in this case simply derived as $D_T = k_B T/f_p$. R_H was determined for the core-shell spherical particles at 465 and 462 nm at 20 and 28°C, respectively. D_T was found to decrease with ρ. Assuming an isochore transformation we can estimate $2a_H$ using the hydrodynamic radius of the core-shell and the aspect ratio from the CLSM analysis, such that $b_H = 2(R_H^3/(b_s/a_s)^2)^{\frac{1}{3}}$. The full lines in Fig. S4C present the expected evolution of D_T for an isochore transformation considering the experimental values of R_H and the dashed lines a fit with R_H as fit parameter. Our values are systematically lower than expected for the prolate core-shell particles, which is surprising as the deformation of the particles would be expected to reduce the swelling of the shell. It does not appear to be related to the quality of the redispersion, which was confirmed by optical microscopy but

rather to some variations of the particles properties during their post-processing into prolates. The best description is there obtained for $R_H = 515$ and 495 nm at 20 and 28°C, respectively.

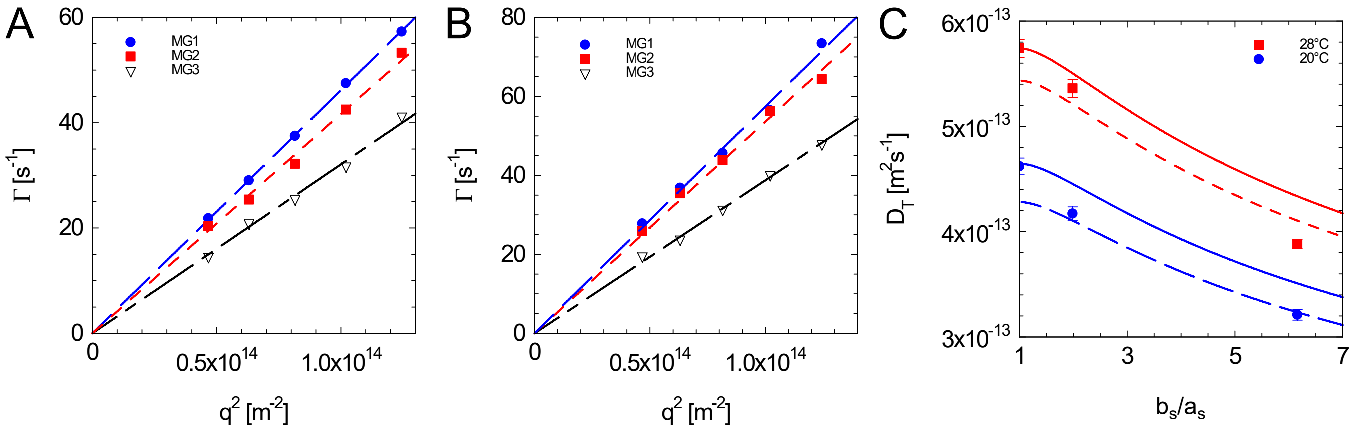

Fig. S4. (A,B) DLS determination of the decayrate Γ as function of q^2 for the different core-shell microgels (γ = 50 to 400%) at 20 °C (A) and 28°C (B). The linear regression enables the determination of the translational diffusion coefficient D_T summarized in (C). (C) Dependence of D_T on the aspect ratio determined by CLSM measurement at 20°C (circles) and 28°C (squares). The full lines indicated the expected dependence for an isochore transformation with the measured hydrodynamic radius of the spherical core-shell particles. The dashed lines are fits for the ellipsoidal microgels considering the original radius of the original spherical particles as fit parameter.

Electrophoretic mobility measurements. The electrophoretic mobility, μ, was determined on dilute dispersions (0.01 wt%) using on a Zetasizer Nano-Z (Malvern) at 20 and 28°C. The measurements indicate that the microgels bare positive charges stemming from some remaining CTAB surfactant and amidine end groups from the 2,2'azobis(2-methylpropionamidine)dihydrochloride (V50) initiator. μ was found to slightly decreases with ρ and with the temperature. Considering that for such cationic microgels most of the charges are located at the surface of the particles (4, 5), the effective charge can be roughly estimate from the DLS measuments as $Q_{eff} \approx f_p\mu \approx \mu k_B T/D_T$ (4). The latter is in the order of 300 - 400e^- for the different measurements. Note that it is about three times larger than "pure" PNIPAM microgels (not core-shell) employed in our former studies, with a comparable hydrodynamic diameter measured at about 450 nm at 20°C (5, 6).

ANS fluorescence analysis. In order to determine the hydrophobicity of the spherical and ellipsoidal microgels, 8-Anilino-1-naphthalenesulfonic acid (ANS, Sigma-Aldrich) was used as a hydrophobic probe (7, 8). Emission spectra were obtained on a Cary Eclipse Fluorescence Spectrophotometer with excitation at 350 nm at 25°C (Fig. S5). The fluorescence result shows that the emission maximum of the three microgels, $MG1$, $MG2$ and $MG3$, are rather close to each other, indicating that the hydrophobicity of the three particles is comparable.

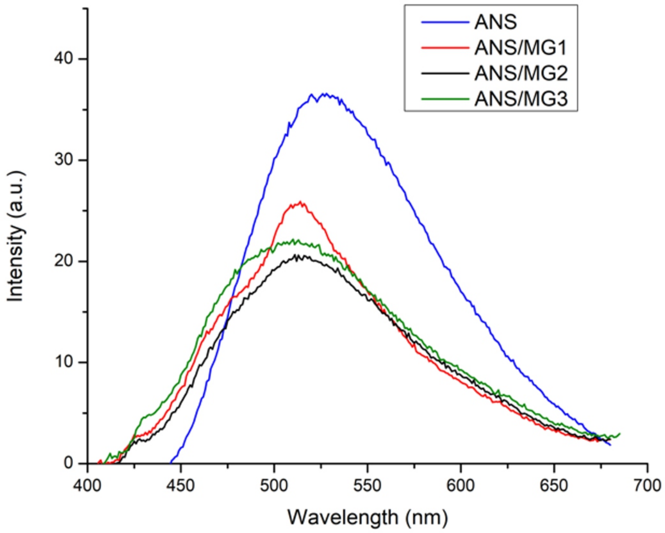

Fig. S5. Fluorescence spectra of ANS (300 μM) recorded for spherical core-shell microgels ($MG1$) and ellipsoidal core-shell microgels ($MG2$ and $MG3$) dispersions in water (0.05 wt%) at 25°C.

Summary of the microgel characterization. γ refer to the applied deformation during the post processing of the core-shell microgels into ellipsoids. $2b_c$, $2a_c$ refer to the long and short axis of the particles measured by TEM in the dried state and ρ_c to their corresponding aspect ratio. $2b$ and $2a$ are the dimensions of the long and short axis measured in aqueous solution at 20 amd 28 °C. D_T refers to the translational diffusion coefficient and μ to the electrophoretic mobility of the core-shell microgels. The effective charge Q_{eff} is estimated from D_T and μ.

Table S1. Summary of the microgel characterization

Systems	core	MG1	MG2	MG3
γ [%]	-	-	50	400
b_c [nm]$^{(1)}$	429 ± 25	494 ± 31	909 ± 67	2472 ± 81
a_c [nm]$^{(1)}$	429 ± 25	494 ± 31	355 ± 24	248 ± 16
$b_c/a_c^{(1)}$	1.0	1.0	2.56 ± 0.17	11.39 ± 1.59
$b_s(20°C)$ [nm]$^{(2)}$	-	830 ± 30	1236 ± 75	2750 ± 97
$a_s(20°C)$ [nm]$^{(2)}$	-	830 ± 30	620 ± 23	446 ± 20
$b_s/a_s(20°C)^{(2)}$	1.0	1.0	2.00 ± 0.08	6.10 ± 0.63
$b_s(28°C)$ [nm]$^{(2)}$	-	830 ± 28	1220 ± 65	2730 ± 95
$a_s(28°C)$ [nm]$^{(2)}$	-	830 ± 28	620 ± 23	450 ± 15
$b_s/a_s(28°C)^{(2)}$	1.0	1.0	1.96 ± 0.08	6.00 ± 0.51
$D_T(20°C)$ $[10^{-13}\ m^2 s^{-1}]^{(3)}$	-	4.62 ± 0.03	4.17 ± 0.17	3.21 ± 0.11
$D_T(28°C)$ $[10^{-13}\ m^2 s^{-1}]^{(3)}$	-	5.74 ± 0.19	5.36 ± 0.18	3.88 ± 0.16
$\mu(20°C)$ $[10^{-8}\ mV^{-1} s^{-1}]^{(4)}$	-	0.56	0.57	0.52
$\mu(28°C)$ $[10^{-8}\ mV^{-1} s^{-1}]^{(4)}$	-	0.76	0.69	0.50
$Q_{eff}(20°C)$ $[e^-]^{(5)}$	-	306	345	409
$Q_{eff}(28°C)$ $[e^-]^{(5)}$	-	334	325	326

- [1] TEM statistical analysis
- [2] CLSM statistical analysis
- [3] DLS measurements
- [4] Electrophoretic mobility measurements
- [5] Estimated from D_T and μ

Adsorption and wrapping of microgels at lipid membrane

Dynamics of microgel adsorption to lipid membranes. Dynamics of MG2 microgel adsorption to DOPC lipid membranes was observed by Nikon A1 Confocal Ti-2 Microscope. The adsorption and rotation of the MG2 microgels at DOPC GUVs was followed over time, as shown in sequential images for three example vesicles (Fig. S6). Representative movies are also provided in Movie S1-S3. The MG2 microgel particles may be oriented in any direction when approaching the membrane. However, as soon as the particles are adsorbed to the membrane they orient with their long axis parallel to the membrane surface. These movies thus imply that irrespectively of the orientation at which the particles approach the membrane, they adsorb with its long axis parallel to the membrane. After adsorption, the particles may further reorient and become deeply wrapped as the final stage structure, but this event fell outside the time frame of the time-resolved experiments.

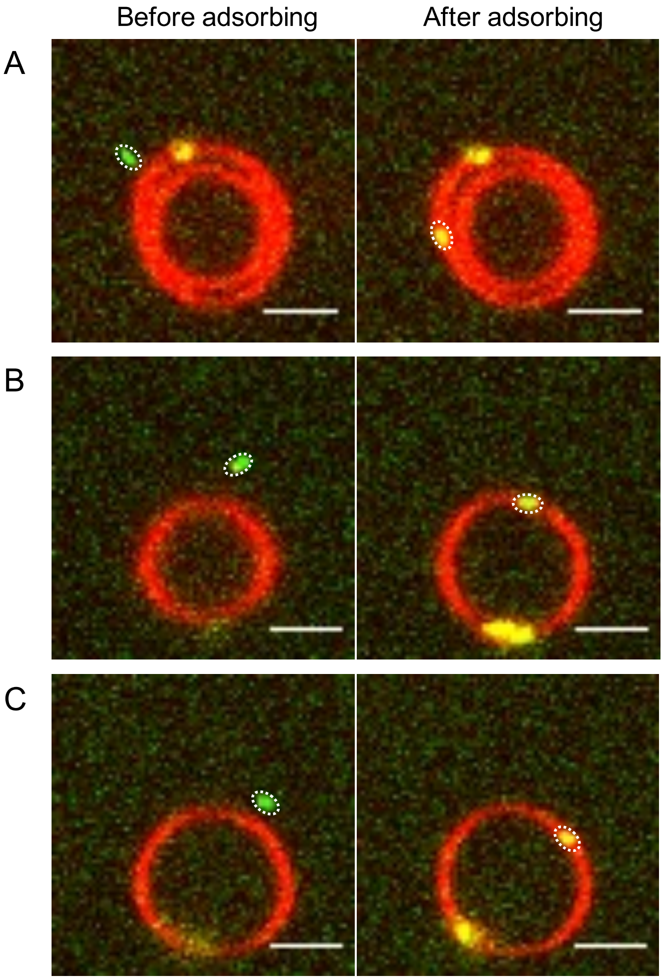

Fig. S6. 2D CLSM images of ellipsoidal MG2 microgels before and after adsorbing on DOPC vesicles. Three examples show that the microgels adsorb on the membrane with side parallel to the membrane even through their initial orientation are different (A, B and C). Temperature: 20 °C. The scale bar: 5 µm.

Microgels associated with GUVs composed of either DOPC, DMPC or DMPC with cholesterol at 28 °C. Additional 2D CLSM micrographs of GUVs composed of either DOPC, DMPC or DMPC/chol decorated by the spherical $MG1$, or the ellipsoidal $MG2$ or $MG3$, microgels are shown in Fig. S7. The different fluorescence channel and their combination are displayed to allow a better visualisation of the membrane deformation. Similarly, CLSM micrographs of ellipsoidal $MG2$ microgels deeply wrapped in the membrane of a GUV composed of DOPC (Fig. S8A), and $MG3$ microgels deeply wrapped in the membrane of a GUV composed of DOPC (Fig. S8B) or DMPC (Fig. S8C).The deep wrapping of the ellipsoidal microgels is clearly evidenced by the deformation of the lipid membrane in the red channel.

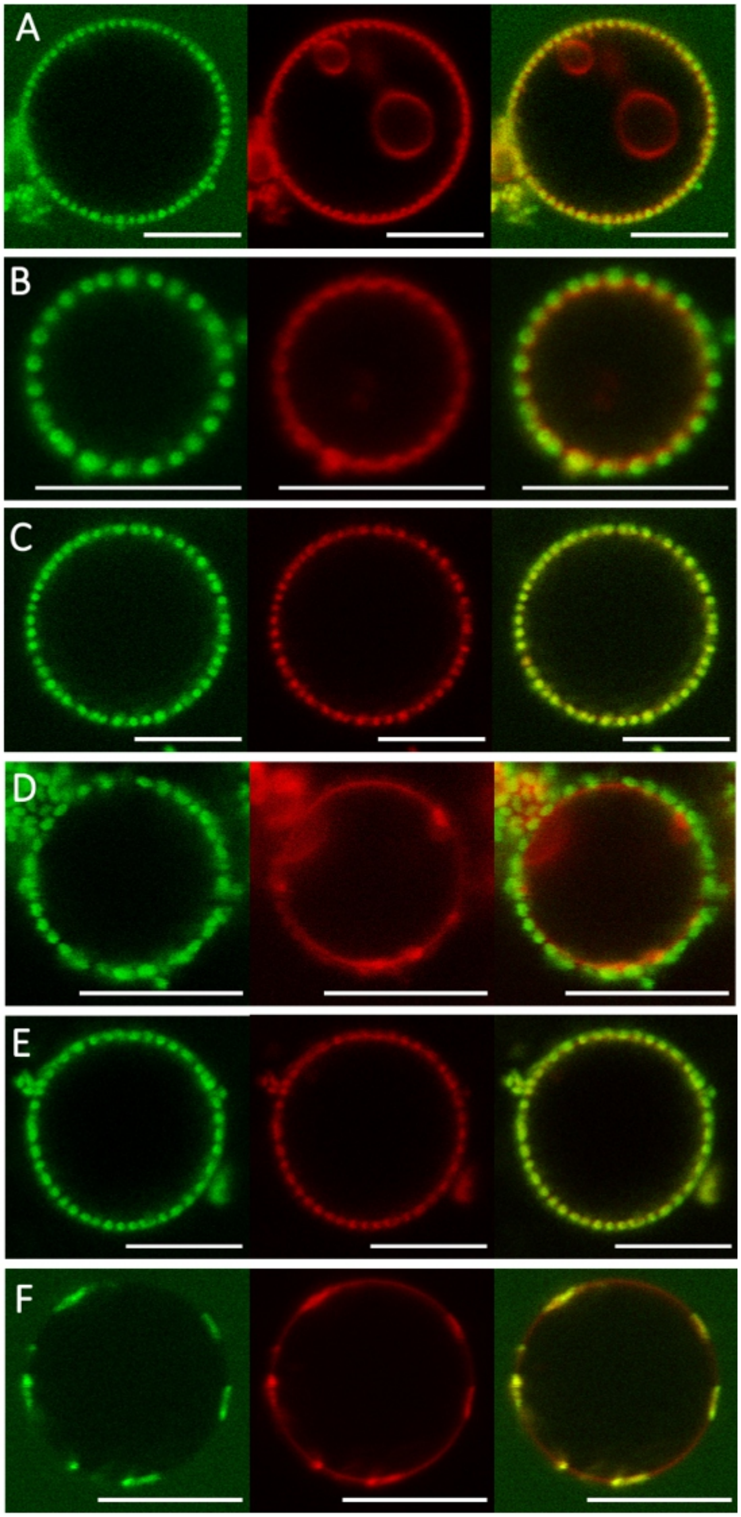

Fig. S7. 2D CLSM micrographs of the spherical $MG1$ microgels adsorbed on DOPC (A), DMPC (B) and DMPC/chol (C) lipid membrane. $MG2$ ellipsoidal microgels adsorbed on DMPC (D) and DMPC/chol (E) lipid membrane, $MG3$ ellipsoidal microgels adsorb on DMPC/chol lipid membrane (F). From left to right, showing green fluorescence presents in microgels, red fluorescence of Liss Rhod PE presents in lipid membranes and overlapping signal of the merged channel. Temperature: 28 ℃. Scale bars: 10 µm.

Microgels associated with DOPC GUV at 20 ℃. Here, we also investigated the spherical and ellipsoidal microgels interacting with the GUV composed of DOPC at 20 °C, 2D and 3D images are shown in Fig. S9 and S10, respectively. The 2D images indicate that the behavior of the microgel particles interacting with DOPC membrane is very similar compared to the results at 28 °C. From the images in Fig. S10, it is clear that the adsorbed spherical $MG1$ microgels form 2D colloidal crystals with

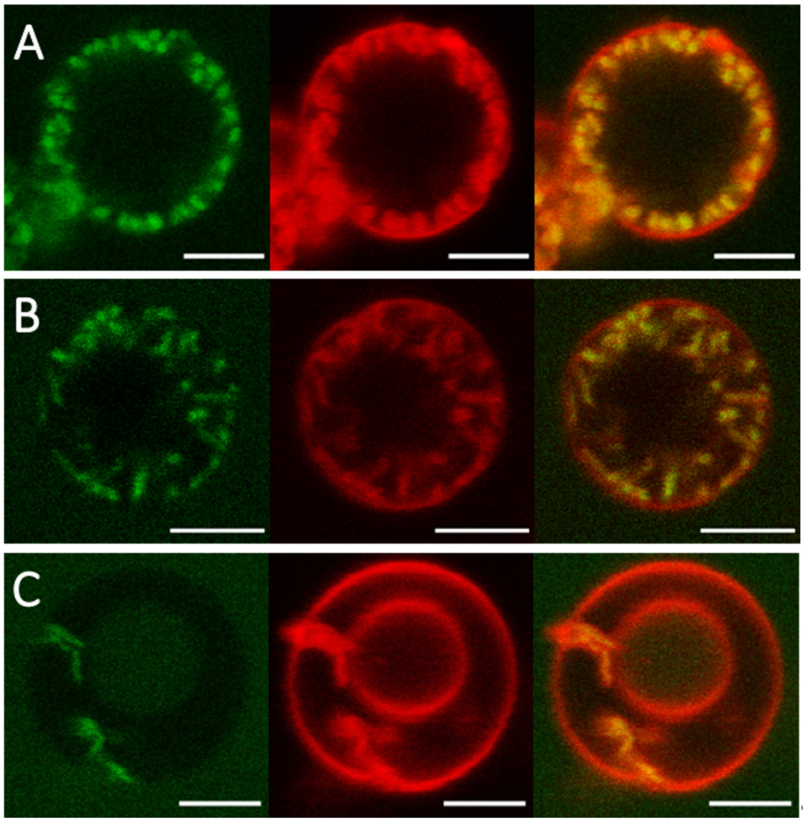

Fig. S8. 2D CLSM micrographs of the ellipsoidal $MG2$ microgels deeply wrapped by DOPC membrane (A) and of the ellipsoidal $MG3$ microgels deeply wrapped by DOPC membrane (B) and DMPC membrane (C). From left to right, showing green fluorescence presents in microgels, red fluorescence of Liss Rhod PE presents in lipid membranes, and overlapping signal of the merged channel. Temperature: 28 ℃. Scale bars: 5 µm.

hexagonal structure on the DOPC membrane, the average center-to-center distance between spherical microgels is 1.19 $\pm$ 0.05 μm. This distance is much larger than the hydrodynamic diameter of the particles pointing that the particles significantly wrap at the membrane and experience some membrane mediated repulsion. These results further indicate that the behavior of the microgels interacting with the GUVs is comparable at 20 and 28 °C.

GUV composed of DOPC, DMPC and cholesterol at different temperature

3D CLSM images of the GUV composed of DOPC, DMPC and cholesterol (DOPC/DMPC/chol, molar ration 7:7:3) at different temperatures are shown in Fig. S11. The DOPC/DMPC/chol GUV is in uniform liquid phase at 28 °C, Fig. S11A. With lowing temperature, as seen in Fig. S11B-D, the GUVs exhibits coexisting liquid phases that include liquid disordered phase and liquid ordered phase. The darker phase is more ordered liquid phase (DMPC/chol-rich) and the brighter phase (DOPC-rich) is less ordered liquid phase. The similar phenomenon was observed in the systems that use similar fluorescent probes in membranes composed of cholesterol and DMPC or cholesterol and DPPC in which the probes partition away from the more ordered state into the less ordered liquid phase (9, 10).

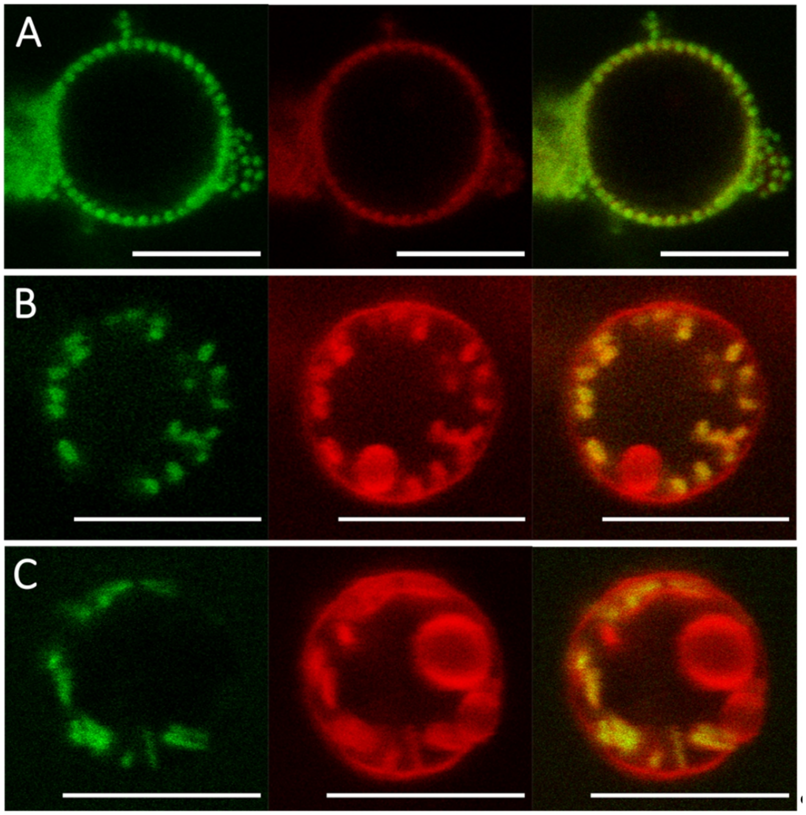

Fig. S9. 2D CLSM micrographs of shallow and deep wrapping of the microgels (green) on DOPC lipid bilayer (red). GUV with adsorbed the spherical $MG1$ (A), the ellipsoidal $MG2$ (B) and $MG3$ (C) microgels. From left to right, showing green fluorescence presents in microgels, red fluorescence of Liss Rhod PE presents in lipid membranes, and overlapping signal of the merged channel. Temperature: 20 ℃. Scale bars: 10 µm.

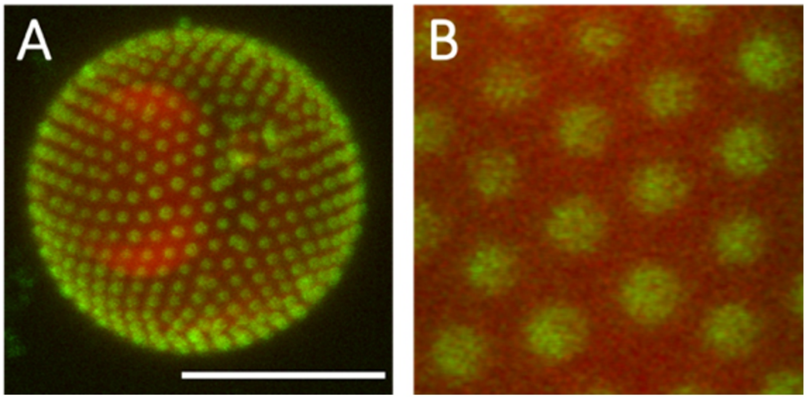

Fig. S10. 3D CLSM images of adsorbed spherical MG1 microgels on the GUVs composed of DOPC (A). The 3D image was reconstructed from confocal z-stack images from the merged channels from microgels (green) and membranes labelled with Liss Rhod PE (red). The corresponding zoom-in image shows the assembling structures of the microgels on the DOPC lipid membrane (B). Temperature: 20 ℃. Scale bar: 10 µm.

Role of membrane tension for the barrier height for deep-wrapping ellipsoidal particles with aspect ratio $b/a = 2$

The influence of the membrane tension σ was investigated for the height of the barrier for the shallow-wrapped to deep-wrapped transition for ellipsoidal particles, see Fig.S12. At low tensions, the barrier for the ellipsoidal particles is higher than for the spherical particles with the same volume due to the high curvature of the particle tip. At high tensions, the barrier for complete-wrapping a spherical particle due to the membrane tension exceeds the barrier that we predict for deep-wrapping the ellipsoidal particle.

Role of the free membrane for wrapping ellipsoidal particles at planar lipid bilayers

The influence of the membrane tension σ was investigated in addition to w as shown in the main text in the discussion of Fig. 6. Figure S13 further illustrates the deformation of the free membrane around the particle for various σ-values.

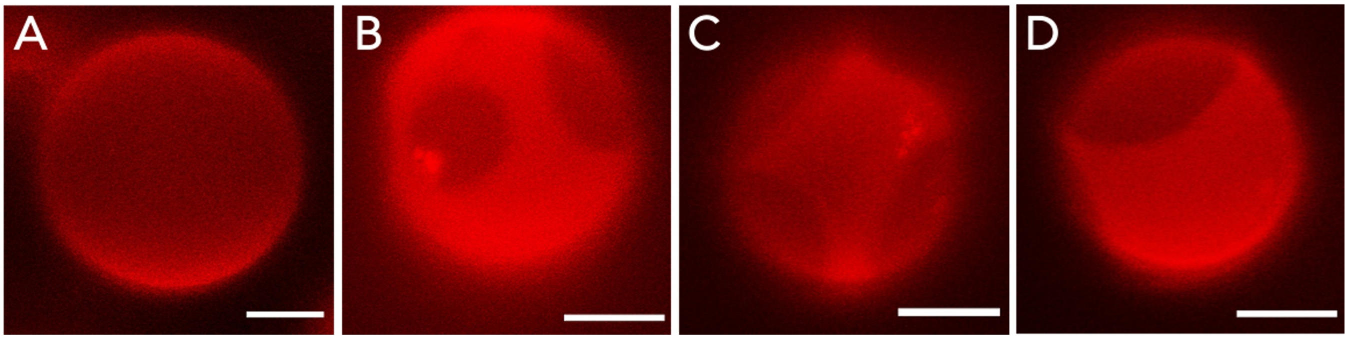

Fig. S11. 3D CLSM images of the GUV composed of DOPC, DMPC and cholesterol (DOPC/DMPC/chol, molar ration 7:7:3), at 28 ℃ (A), 17 ℃ (B), 16.5 ℃ (C) and 16 ℃ (D). Scale bars: 5 µm.

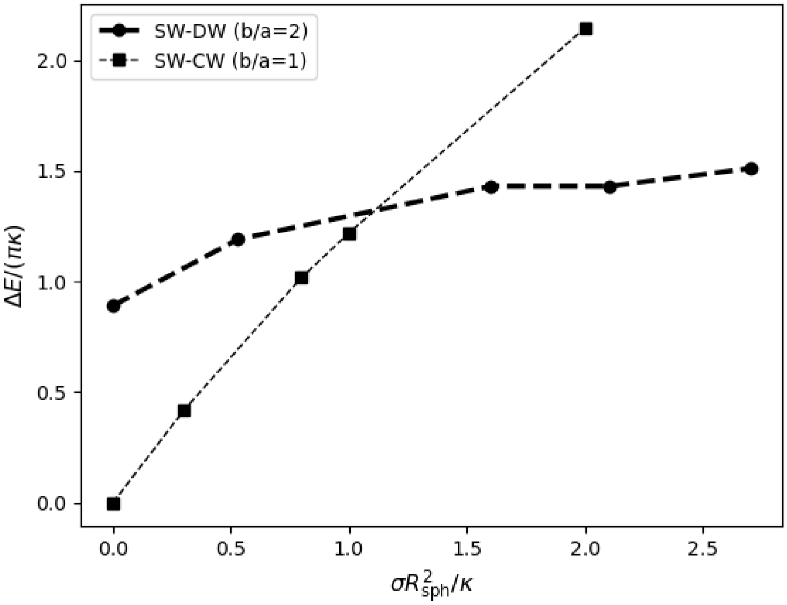

Fig. S12. Energy barrier for wrapping an ellipsoidal particle with aspect ratio 2 equal volume and a spherical particle (data for the spherical particle reused from Ref. (11)).

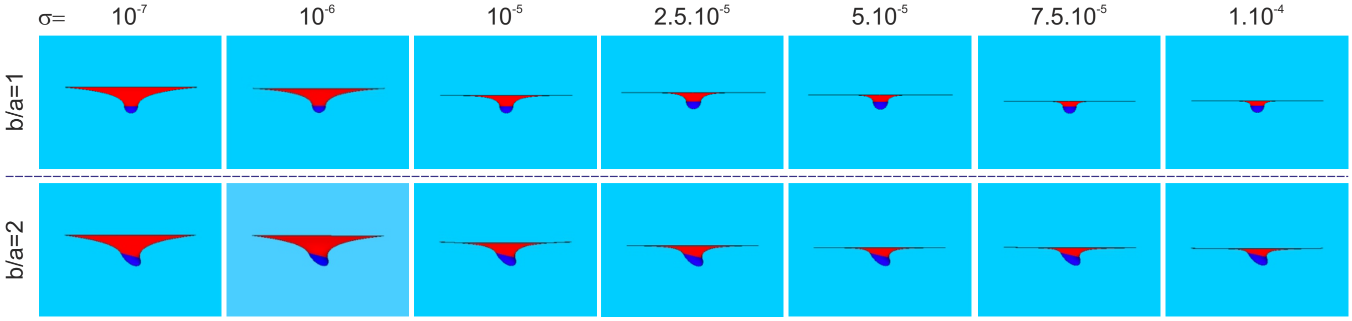

Fig. S13. Simulation snapshots of the spherical (A) and ellipsoidal (B) particle wrapping at initially planar lipid-bilayer membrane at various membrane tension. The volume of the particles is V_0=0.31 μm^3, the ellipsoidal particle has an aspect ratio $b/a = 2$ and a tilt angle of 45° and the membrane bending rigidity is $\kappa = 20 k_B T$.

Elongated nanoparticles at vesicles

Wrapping of elongated nanoparticles at vesicles includes the local curvature of the particle surface and elastic deformation-energy costs of the lipid bilayer (11, 12). For spherocylinders, both aspects can be discussed on the basis of analytical expressions for the energy contributions; we restrict ourselves to rocket orientation, such that we can exploit cylindrical symmetry. Plots for the parameters relevant for the experiments are shown in Fig. S14.

We assume an initially spherical vesicle of area $A_\mathrm{v} = 4\pi R_\mathrm{v}^2$ immersed in a solvent with osmotic concentration c_v. The

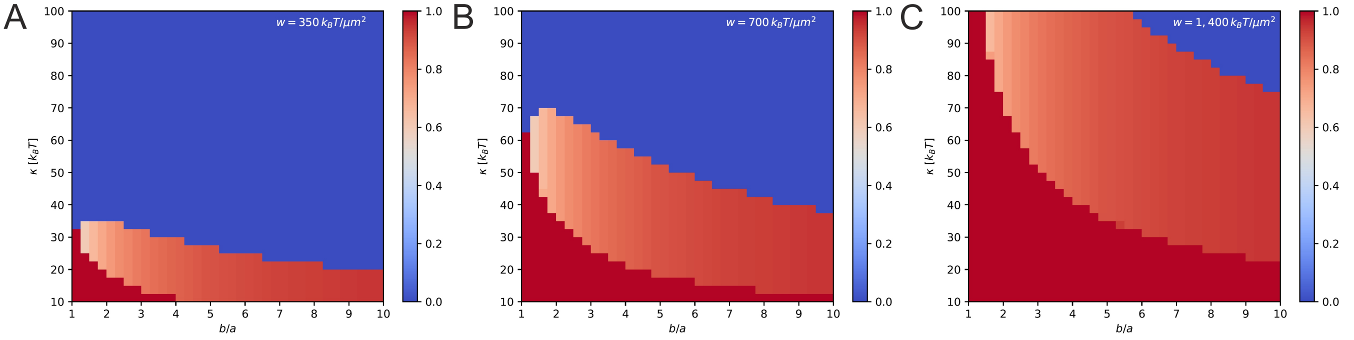

Fig. S14. Wrapping diagram for spherocylindrical particles with aspect ratios $1 \le b/a \le 10$ at initially spherical vesicles with radius $R = 5\,\mu\mathrm{m}$ for membrane bending rigidities $10 \le \kappa \le 100$. The lines indicate half-wrapping of the particles for adhesion strengths (A) $w = 350\,k_BT/\mu\mathrm{m}^2$, (B) $700\,k_BT/\mu\mathrm{m}^2$, and (C) $1400\,k_BT/\mu\mathrm{m}^2$ and the parameters in Tab. S2.

membrane area

$$A_{\mathrm{ad}} = \frac{4\ 2^{2/3}\pi f_{\mathrm{w}} b R_{\mathrm{p}}^2}{3b-a} \qquad [2]$$

adhered to a particle increases with increasing wrapping fraction of the spherocylinder, where the total surface area of the isochoric particles increases with increasing aspect ratio. Because of the fixed total membrane area the free membrane area for N particles that are equally wrapped decreases as $A_{\mathrm{free}} = A_{\mathrm{v}}^2 - NA_{\mathrm{ad}}$. Similarly, the radius of the mother vesicle is

$$R_{\mathrm{v}'} = \frac{1}{2}\sqrt{N_{\mathrm{p}}A_{\mathrm{sc,r}}^2 - \frac{4\ 2^{2/3} f_{\mathrm{w}} N_{\mathrm{p}} R_{\mathrm{p}}^2}{(3b/a-1)^{2/3}} - \frac{4\ 2^{2/3}(b/a-1) f_{\mathrm{w}} N_{\mathrm{p}} R_{\mathrm{p}}^2}{(3b/a-1)^{2/3}} + 4R_{\mathrm{v}}^2} \qquad [3]$$

with

$$A_{\mathrm{sc,r}} = \begin{cases} \frac{2\sqrt[3]{2}R_{\mathrm{p}}\sqrt{b/af_{\mathrm{w}}(1-b/af_{\mathrm{w}})}}{\sqrt[3]{3b/a-1}} & f_{\mathrm{w}} \le \frac{1}{2b/a} \\ \frac{\sqrt[3]{2}R_{\mathrm{p}}}{\sqrt[3]{3b/a-1}} & \frac{1}{2b/a} \le f_{\mathrm{w}} < 1 - \frac{1}{2b/a} \\ \frac{2\sqrt[3]{2}R_{\mathrm{p}}\sqrt{-b/a(f_{\mathrm{w}}-1)(b/a(f_{\mathrm{w}}-1)+1)}}{\sqrt[3]{3b/a-1}} & f_{\mathrm{w}} \ge 1 - \frac{1}{2b/a}\,. \end{cases} \qquad [4]$$

This leads to the effective volume

$$V' = \frac{4}{3}\pi R_{\mathrm{v}'}^3 - N_{\mathrm{p}}(\mathrm{VcutNpscr}(\mathrm{Rv}, \mathrm{Rp}, \mathrm{bOa}, \mathrm{fw}, \mathrm{Np}) + \mathrm{Vadscr}(\mathrm{Rp}, \mathrm{bOa}, \mathrm{fw})) \qquad [5]$$

decrease with increasing wrapping fraction. This yields the bending energy

$$E_{\mathrm{b}} = N_{\mathrm{p}}E_{\mathrm{b,ad}} + 2\kappa A_{\mathrm{f}}\left(\frac{(3b/a-1)^{1/3}}{2^{1/3}R_{\mathrm{v}'}}\right)^2, \qquad [6]$$

where

$$E_{b,ad} = \begin{cases} \frac{4\left(2^{2/3}b/af_{\mathrm{w}}\pi R_{\mathrm{p}}{}^2\right)2\kappa\left(\frac{\sqrt[3]{3b/a-1}}{\sqrt[3]{2}R_{\mathrm{p}}}\right)^2}{(3b/a-1)^{2/3}} & f_{\mathrm{w}} \le \frac{1}{2b/a} \\ \frac{2\left(2\ 2^{2/3}\pi R_{\mathrm{p}}^2\right)\kappa\left(\frac{\sqrt[3]{3b/a-1}}{\sqrt[3]{2}R_{\mathrm{p}}}\right)^2}{(3b/a-1)^{2/3}} + 2\left(\frac{4\ 2^{2/3}b/af_{\mathrm{w}}\pi R_{\mathrm{p}}^2}{(3b/a-1)^{2/3}} - \frac{2\ 2^{2/3}\pi R_{\mathrm{p}}^2}{(3b/a-1)^{2/3}}\right)\kappa\left(\frac{\sqrt[3]{3b/a-1}}{2\sqrt[3]{2}R_{\mathrm{p}}}\right)^2 & \frac{1}{2b/a} \le f_{\mathrm{w}} < 1 - \frac{1}{2b/a} \\ 2\left(\frac{4\ 2^{2/3}b/af_{\mathrm{w}}\pi R_{\mathrm{p}}^2}{(3b/a-1)^{2/3}} - \frac{4\ 2^{2/3}(b/a-1)\pi R_{\mathrm{p}}^2}{(3b/a-1)^{2/3}}\right)\kappa\left(\frac{\sqrt[3]{3b/a-1}}{\sqrt[3]{2}R_{p}}\right)^2 + \frac{2\left(4\ 2^{2/3}(b/a-1)\pi R_{\mathrm{p}}^2\right)\kappa\left(\frac{\sqrt[3]{3b/a-1}}{2\sqrt[3]{2}R_{\mathrm{p}}}\right)^2}{(3b/a-1)^{2/3}} & f_{\mathrm{w}} \ge 1 - \frac{1}{2b/a} \end{cases} \qquad [7]$$

is the bending energy of the membrane adhered to one particle, which is a piecewise function for the spherical caps and the cylindrical barrel. The second term describes the change of the bending energy of the free membrane of the vesicle. Here, A_{f} is the area of the free membrane. The adhesion energy is $E_{\mathrm{ad}} := -N_{\mathrm{p}}A_{\mathrm{ad}}w$ with the adhesion strength w. The total energy

$$E_{\mathrm{tot}} = E_{\mathrm{b}} + E_{\mathrm{ad}} \qquad [8]$$

is the sum of the bending energy and the adhesion energy.

Tip-wrapping elongated nanoparticles: membrane holes vs. blisters

Particle wrapping at lipid-bilayer membranes is favored by the energy gain through the particle-membrane adhesion, and hindered by the energy costs for deforming the membrane. These two processes are characterized by the particle-membrane adhesion strength w and the membrane bending rigidity κ. Wrapping of highly elongated nanoparticles is particularly disfavored by the need to strongly deform the membrane next to the tips (11). However, there are two possibilities to avoid such unfavourable shapes: (i) a hole in the membrane at the tip location, which generates an open membrane boundary with a penalty due to the line tension of the boundary, or (ii) the formation of a membrane "blister", which encloses the tip. In order to see which of the two options is favorable, we calculated and compare the corresponding energies. We consider wrapping of prolate ellipsoidal nanoparticles with aspect ratio b/a and cones that mimic a particle with a sharp tip, see Fig. S15.

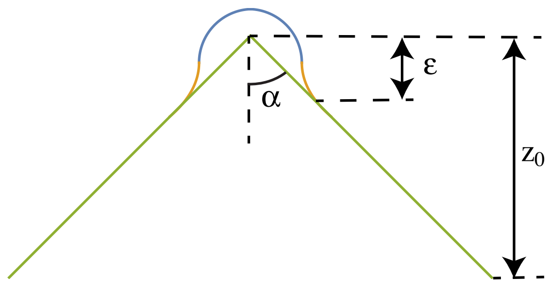

Fig. S15. Wrapping of cones. The membrane either forms a blister or a hole at a distance ϵ from the tip; a blister is modeled as a catenoid connected to a spherical cap. The deformation energy is calculated up to a height z_0.

For a blister at the tip, the bending energy is at least a cap formed by a half sphere, and at most an entire sphere,

$$4\pi\kappa \leq E_{\mathrm{c}} < 8\pi\kappa\,. \qquad [9]$$

Here, only the bending energy of the blister has to be taken into account for because the neck, which connects the blister to the adhered part of the membrane, can be assumed having a catenoidal shape with vanishing bending energy (13). For simplicity, we will work with the bending energy for a half-spherical blister, $E_{\mathrm{c}} = 4\pi\kappa$, in the following. The total energy for the formation of a blister at the tip is the sum of the bending energy E_{b} of the membrane attached to the particle, the blister energy E_{c}, and the adhesion energy E_{ad},

$$E_{\mathrm{tot,cap}} = E_{\mathrm{b}} + E_{\mathrm{c}} + E_{\mathrm{ad}}\,. \qquad [10]$$

Holes have been observed experimentally at the vertices of icosahedral lipid vesicles (14). Membrane binding to a highly curved tip of an ellipsoid may lead to the formation of holes as well. Here, a line-energy cost E_{l} for the formation of the hole occurs. The total energy for the formation of a hole at the tip is therefore

$$E_{\mathrm{c}} = E_{\mathrm{b}} + E_{\mathrm{l}} + E_{\mathrm{ad}}\,. \qquad [11]$$

Wrapping energies for ellipsoidal nanoparticles. The surface of the ellipsoid with the short axis a and long axis b is parametrized as $\mathbf{r}(z,\phi) = (a(b^2-z^2)^{1/2}\cos\phi, a(b^2-z^2)^{1/2}\sin\phi, z)$; bending energy costs can be calculated analytically (15). The mean curvature is

$$H_{\mathrm{ell}}(z) = \frac{a^2\left(b^3\,(b-z)\,(b+z) + a^2 b\left(b^2+z^2\right)\right)}{2b^2\left(a^2\left(b^2-z^2+a^2z^2/b^2\right)\right)^{3/2}}\,, \qquad [12]$$

and the bending energy for an ellipsoid wrapped up to detachment length ϵ is

$$\begin{aligned} E_{\mathrm{b}}(\epsilon) &= \frac{4\pi\kappa a}{b}\int_0^{b-\epsilon} dz\,\sqrt{b^2-z^2}\sqrt{1+\left(\frac{az}{b\sqrt{b^2-z^2}}\right)^2}\left(\frac{a^2\left(b^3(b-z)(b+z)+a^2b(b^2+z^2)\right)}{x}2b^2\left(a^2(b^2-z^2+a^2z^2/b^2)\right)^{3/2}\right)^2 \\ &= \pi\kappa\int_0^{b-\epsilon} dz\,\frac{b^3\sqrt{(b-z)(b+z)}\sqrt{1+\left(az/(b\sqrt{b^2-z^2})\right)^2}\left(b^4-b^2z^2+a^2b(b^2+z^2)\right)^2}{a(b^4+a^2z^2-b^2z^2)^3}\,. \end{aligned} \qquad [13]$$

For a hole at the tip, the line energy is

$$E_{\mathrm{l}}(\epsilon) = 2\pi\lambda\frac{a\sqrt{b^2-(b-\epsilon)^2}}{b}\,. \qquad [14]$$

The adhesion energy is

$$E_{\mathrm{ad}}(\epsilon) = -\frac{2\pi w a}{b}\int_0^{b-\epsilon} dz\,\sqrt{b^2-z^2}\sqrt{1+\left(\frac{az}{b\sqrt{b^2-z^2}}\right)^2}\,. \quad [15]$$

Figure S16 shows the total energy and the energy contributions for the formation of a hole and of a blister for particles with aspect ratios $b/a = 2$ and 10 as function of the detachment length ϵ from the tip. The global minimum of the total energy determines whether the ellipsoid is complete-wrapped or partial-wrapped with the membrane detached at the tip. With increasing detachment length ϵ both bending-energy cost and adhesion-energy gain decrease. However, for hole formation the line energy increases with the radius of the hole. For blister formation, we assume an additional, constant energy cost E_{c} for all finite ϵ. For the parameters chosen, a blister at the tip is stable for $b/a \gtrsim 8$ when the minimum of the energy at finite detachment length ϵ is lower than the complete-wrapped ellipsoid, whereas a pore forms only for aspect ratios $b/a \geq 52$.

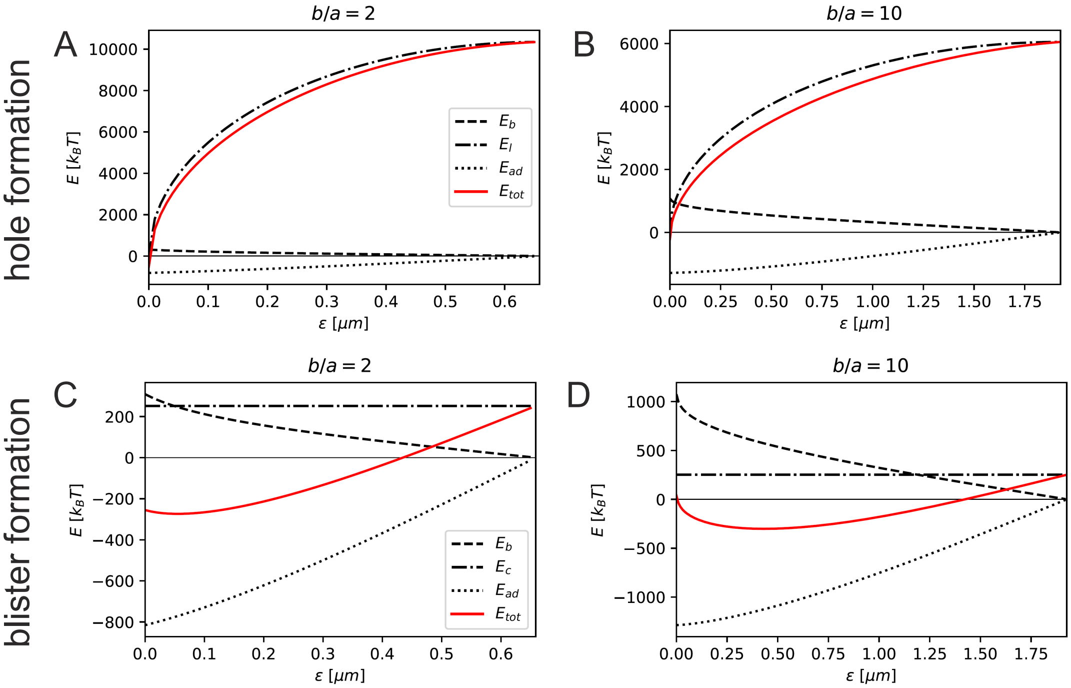

Fig. S16. Wrapping energies for half ellipsoids with aspect ratios $b/a = 2$ and $b/a = 10$ as function of the detachment length ϵ from the tip for (a-b) formation of a hole, and (c-d) formation of a blister. The figure shows the total energy E_{tot}, as well as the bending energy E_{b}, the line tension for the hole E_{l}, the blister/cap energy E_{c}, and the adhesion energy E_{ad} for the parameters in Tab. S2. The bending energy of a half-spherical cap is assumed as energy cost for the formation of a blister.

Wrapping phases for ellipsoids. For the tip-wrapping of ellipsoidal particles, we predict non-wrapped and complete-wrapped states, as well as pore formation and–for sufficiently high aspect ratios–blister formation at the tips. Figure S17 shows wrapping phase diagrams for particles with $b/a = 2$, 10, and 50. For particles with $b/a = 2$ we predict a non-wrapped-complete-wrapped transition for sufficiently high line tensions that suppress the formation of a hole. For small line tensions pore formation at the tips occurs, which somewhat corresponds to shallow-wrapped states in submarine orientation for free ellipsoidal particles at membranes (11, 12)–a state that is prohibited by construction in our tip-wrapping calculations. For particles with $b/a = 10$ and 50 the bending energy costs at the tips are sufficiently high to allow the system to form blisters for intermediate adhesion strengths. The blister-regime replaces the direct transition between non-wrapped and complete-wrapped states that is found for high line tensions for particles with $b/a = 2$.

Table S2. Parameters for wrapping calculations.

Quantity	Value	References
bending rigidity κ	$20\,k_BT$	
line tension λ	$5\times10^3\,k_BT/\mu\mathrm{m}$	(16)
adhesion strength w	$700\,k_BT/\mu\mathrm{m}^2$	(6)
radius of isochorus spherical particle R	$415\,\mathrm{nm}$	
aspect ratios of ellipsoidal particles b/a	2; 10	

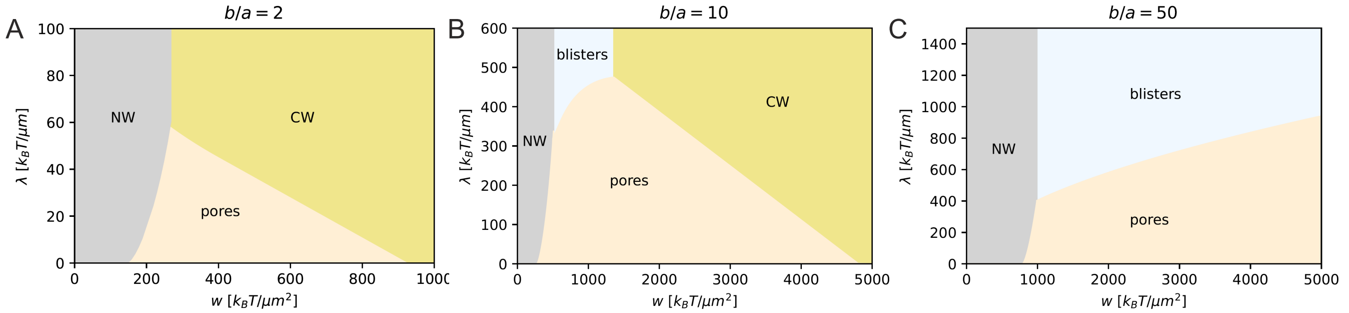

Fig. S17. Wrapping phase diagrams for ellipsoidal particles with various particle-membrane adhesion strengths w and line tensions λ for the parameters in Tab. S2. Non-wrapped (NW) and complete-wrapped (CW) states are indicated along with states with pores and blisters at the tips for particles with aspect ratios (a) $b/a = 2$ and (b) $b/a = 10$.

Wrapping energies for cones. The surface of a cone with an opening angle γ is parametrized as $\mathbf{r}(z,\phi) = (\gamma z, \phi)$. The mean curvature is

$$H_{\mathrm{cone}}(z) = \frac{1}{2z\gamma\sqrt{1+\gamma^2}}\,, \qquad [16]$$

which yields the bending energy

$$E_{\mathrm{b}}(\epsilon) = \frac{\pi\kappa}{\gamma\sqrt{1+\gamma^2}} \ln \frac{z_{\mathrm{max}}}{\epsilon} \qquad [17]$$

Here, z_{max} is the size of the cone; changing z_{max} corresponds to adding or subtracting constant terms to the bending energy and thus does not affect tip-wrapping predictions. For a hole at the tip, the line energy is

$$E_{\mathrm{l}}(\epsilon) = 2\pi\lambda\gamma\epsilon\,. \qquad [18]$$

The adhesion energy is

$$E_{\mathrm{ad}}(\epsilon) = -w\pi\gamma\sqrt{1+\gamma^2}(z_{\mathrm{max}}^2 - \epsilon^2)\,. \qquad [19]$$

Figure S18 shows the relevant energies of the system. Unlike for ellipsoids, the bending energy diverges at the conical tip, such that the theory always predicts hole or a blister formation–although at different distances from the tip. Here, it has to be kept in mind that the Helfrich model applies for small mean curvatures.

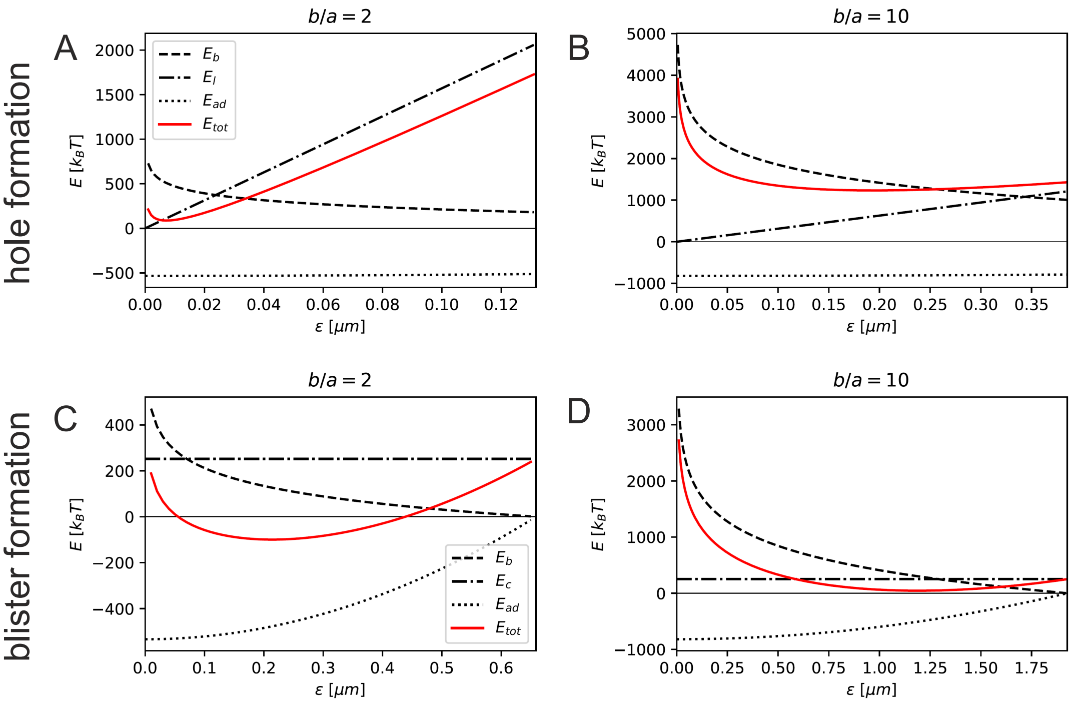

Fig. S18. Wrapping energies for cones with $\gamma = b/a$ that correspond to ellipsoids with aspect ratios $b/a = 2$ and $b/a = 10$ as function of the detachment length ϵ from the tip for (A,B) formation of a hole, and (C,D) formation of a blister. The height of the cone is chosen to be the long axis of the ellipsoid. The figure shows the total energy E_{tot}, as well as the bending energy E_{b}, the line tension for the hole E_{l}, the blister/cap energy E_{c}, and the adhesion energy E_{ad} for the parameters in Tab. S2. The bending energy of a half-spherical cap is assumed as energy cost for the formation of a blister.

Wrapping phases for cones. The bending energy of cones diverges at the tip, therefore complete-wrapped states cannot be observed independent of the value for the particle-membrane adhesion strength. Instead, we predict the formation of a hole at the tip for small line tensions and blister formation for large line tensions, see Fig. S19. This finding qualitatively agrees with our predictions for wrapping the tips of ellipsoidal particles in Fig. S16. For a cone with a larger opening angle γ, the transition to blister formation occurs at higher adhesion strengths w. Interestingly, the phase boundaries between hole and blister formation state scale as $\lambda \sim w^{1/2}$.

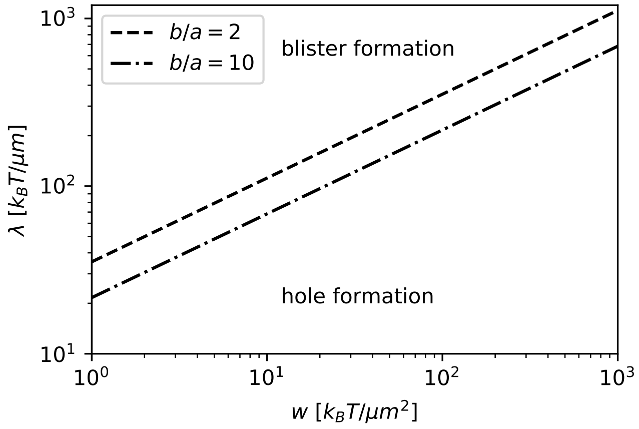

Fig. S19. Wrapping phase diagram for cones for various particle-membrane adhesion strengths w and line tensions λ for the parameters in Tab. S2. The lines indicate the phase boundaries that separate the regimes where pore formation and blister formation are predicted.

Supporting movies

Movie S1. The adsorption and rotation of $MG2$ microgels at DOPC GUVs was followed over time.

Movie S2. The adsorption and rotation of $MG2$ microgels at DOPC GUVs was followed over time.

Movie S3. The adsorption and rotation of $MG2$ microgels at DOPC GUVs was followed over time.

References

1. JJ Crassous, et al., Field-induced assembly of colloidal ellipsoids into well-defined microtubules. *Nat. Commun.* **5**, 1–7 (2014).
2. F Perrin, Mouvement brownien d'un ellipsoïde (ii). rotation libre et dépolarisation des fluorescences. translation et diffusion des molécules ellipsoïdales. *J. Phys. Radium* **1**, 1–11 (1936).
3. I Martchenko, H Dietsch, C Moitzi, P Schurtenberger, Hydrodynamic properties of magnetic nanoparticles with tunable shape anisotropy: prediction and experimental verification. *J. Phys. Chem. B* **115**, 14838–14845 (2011).
4. T López-León, JL Ortega-Vinuesa, D Bastos-González, A Elaïssari, Cationic and anionic poly (n-isopropylacrylamide) based submicron gel particles: Electrokinetic properties and colloidal stability. *J. Phys. Chem. B* **110**, 4629–4636 (2006).
5. A Mihut, B Stenqvist, M Lund, P Schurtenberger, J Crassous, Assembling oppositely charged lock and key responsive colloids: A mesoscale analog of adaptive chemistry. *Sci. Adv.* **3**, e1700321 (2017).
6. M Wang, et al., Assembling responsive microgels at responsive lipid membranes. *Proc. Natl. Acad. Sci. U. S. A.* **116**, 5442–5450 (2019).
7. K Fujimoto, Y Nakajima, M Kashiwabara, H Kawaguchi, Fluorescence analysis for thermo-sensitive hydrogel microspheres. *Polym. Int.* **30**, 237–241 (1993).
8. O Gasymov, B Glasgow, Ans fluorescence: potential to augment the identification of the external binding sites of proteins. *Biochim. Biophys. Acta* **1774**, 403–411 (2007).
9. LM Loura, A Fedorov, M Prieto, Fluid–fluid membrane microheterogeneity: a fluorescence resonance energy transfer study. *Biophys. J.* **80**, 776–788 (2001).
10. SL Veatch, SL Keller, Separation of liquid phases in giant vesicles of ternary mixtures of phospholipids and cholesterol. *Biophys. J.* **85**, 3074–3083 (2003).
11. S Dasgupta, T Auth, G Gompper, Wrapping of ellipsoidal nano-particles by fluid membranes. *Soft Matter* **9**, 5473–5482 (2013).
12. S Dasgupta, T Auth, G Gompper, Shape and orientation matter for the cellular uptake of nonspherical particles. *Nano Lett.* **14**, 687–693 (2014).
13. T Auth, G Gompper, Budding and vesiculation induced by conical membrane inclusions. *Phys. Rev. E* **80**, 031901 (2009).
14. M Dubois, et al., Self-assembly of regular hollow icosahedra in salt-free catanionic solutions. *Nature* **411**, 672–675 (2001).
15. J Agudo-Canalejo, Engulfment of ellipsoidal nanoparticles by membranes: full description of orientational changes. *J. Condens. Matter Phys.* **32**, 294001 (2020).
16. SA Akimov, et al., Pore formation in lipid membrane i: Continuous reversible trajectory from intact bilayer through hydrophobic defect to transversal pore. *Sci. Rep.* **7**, 1–20 (2017).
